# Partner Bereavement and Detection of Dementia: A UK-Based Cohort Study Using Routine Health Data

**DOI:** 10.3233/JAD-190571

**Published:** 2019-11-12

**Authors:** Harriet J. Forbes, Angel Y.S. Wong, Caroline Morton, Krishnan Bhaskaran, Liam Smeeth, Marcus Richards, Sigrun A.J. Schmidt, Sinéad M. Langan, Charlotte Warren-Gash

**Affiliations:** a London School of Hygiene & Tropical Medicine, London, UK; b MRC Unit for Lifelong Health and Ageing at UCL, University College London, London, UK; c Department of Clinical Epidemiology, Aarhus University Hospital, Aarhus, Denmark; d Department of Dermatology, Aarhus University Hospital, Aarhus, Denmark

**Keywords:** Bereavement, Clinical Practice Research Datalink, dementia, diagnosis, epidemiology

## Abstract

**Background:**

In the UK, an estimated one third of people with dementia have not received a diagnosis. Good evidence suggests that dementia risk is increased among widowed individuals; however, it is not clear if they are being diagnosed in routine primary care.

**Objective:**

This study aimed to investigate if bereavement influenced the probability of having received a dementia diagnosis.

**Methods:**

A population-based cohort study using UK electronic health records, between 1997 and 2017, among 247,586 opposite-sex partners. Those experiencing partner bereavement were matched (age, sex, and date of bereavement) to a non-bereaved person living in a partnership. Multivariate cox regression was performed.

**Results:**

Partner bereavement was associated with an increased risk of receiving a diagnosis of dementia in the first three months (hazard ratio (HR) 1.43, 95% CI 1.20–1.71) and first six months (HR 1.24, 95% CI 1.09–1.41), while there was a small reduced risk of getting a dementia diagnosis over all follow-up (HR 0.94, 95% CI 0.89–0.98).

**Conclusions:**

Partner bereavement appears to lead to a short-term increased risk of the surviving partner receiving a diagnosis of dementia, suggesting that bereavement unmasks existing undiagnosed dementia. Over the longer term, however, bereaved individuals are less likely to have a diagnosis of dementia in their health records than non-bereaved individuals.

## INTRODUCTION

An estimated 850,000 people are currently living with dementia in the UK, with around 34% of dementia patients believed to be undiagnosed [1, 2]. A timely diagnosis for people living with dementia is essential to enable patients and their carers to receive necessary care and support. Many governments, including the UK, are committed to increasing the number of dementia patients who have received a formal diagnosis [2]. Although the age-specific incidence is decreasing in the UK [3], the burden of dementia is rising as the population ages [4], impressing the need for better detection of dementia cases.

A comprehensive meta-analysis of seven cohort studies from Europe and North America demonstrates an increased dementia risk among widowed compared to married individuals (pooled relative risk, 1.10, 95% CI: 1.05–1.28) [5]. Possible reasons include shared dementia risk factors between partners (such as smoking habits), acute psychological distress causing direct neural damage [6], and behavioral changes following bereavement, such as poor diet, poorer medication coverage [7], and social isolation.[8, 9] However, it is not clear if widowed individuals are receiving a dementia diagnosis in routine clinical care.

Studies in this area to date have investigated whether bereavement may increase the long-term risk of developing dementia, thereby assessing whether bereavement is a potential causal factor. Our study instead aimed to investigate if a recent partner loss affects the risk of being diagnosed with dementia. This has not be assessed in a UK population-based setting [5]. We therefore conducted a population-based study using standardized approaches in UK general practice to study the relationship between partner bereavement and first diagnosis of dementia.

## METHODS

### Study design and setting

We conducted a population-based, matched cohort study to investigate the relationship between partner bereavement and receiving a first diagnosis of dementia. We used data from the UK Clinical Practice Research Datalink (CPRD), as well as linked Hospital Episodes Statistics (HES) data, Office of National Statistics (ONS) death data, and Index of Multiple Deprivation (IMD) data. CPRD is a routinely collected database of anonymized primary care records from around 9% of the UK population [10, 11]. Data include diagnoses (coded using Read codes), prescriptions (coded using British National Formulary codes), and referrals. Approximately 80% of CPRD practices in England have linked data from other sources. HES data contain all NHS admissions in England since 1997, including diagnoses (coded using International Classification of Diseases, 10th version). IMD data provide quintiles of deprivation based on the practice postcode. All code lists are here: DOI: https://doi.org/10.17037/DATA.00001003).

### Participants

The study population included all persons aged 40 years or older who experienced partner bereavement, and matched persons with a living partner.

The algorithm to identify partners has been described elsewhere [12] (full details in the Supplementary Material). Briefly, partners were persons within the same household, of the opposite sex, with an age gap of ≤10 years and with no younger adult in the same household within 15 years of either member of the couple. Death of one member of the couple was identified and the surviving partner included in the bereaved (exposed) cohort. Date of partner death was considered the index date (obtained from ONS data where available, otherwise obtained from CPRD). Persons in the comparison cohort had to be alive and have a partner, but without ever experiencing previous partner bereavement on the index date of the randomly matched (on age at bereavement and sex, without replacement) exposed person. Unexposed persons could move into the exposed group if they experienced a bereavement during follow-up. We excluded participants with <2 years eligible CPRD follow-up prior to index date (to ensure adequate capture of pre-existing dementia risk factors) and those with diagnosed dementia prior to index date.

### Follow-up

Follow-up for exposed and matched unexposed persons began at index date and ended at the earliest of either study end (31 July 2017), death, transfer out of practice, practice last collection date, or first dementia diagnosis. Patients contributing at least one day of follow-up were included. If either an unexposed person or their partner left the practice, both were censored from that date.

### Variables

#### Exposure

Partner bereavement is described above. Our secondary exposures were predicted risk of partner death (which aimed to capture whether death was anticipated) and living alone during the bereavement period (ascertained using family number to ascertain the number of people living in the household). We calculated an age-adjusted Charlson Comorbidity Index (CCI) score at one month before death for partners of the exposed (bereaved) cohort [13], to classify the predicted risk of partner death as low, intermediate, or high. For both secondary exposures, the baseline group remained as those not experiencing partner death.

#### Outcomes

The primary outcome was incident diagnosed dementia, identified in CPRD and HES data. If dementia was recorded in both data sources, the earliest date of diagnosis was taken. Dementia often has a mixed pathology and specific diagnoses are infrequently recorded in health records. We therefore used a broad definition that incorporates codes for vascular dementia, Alzheimer’s disease, and unspecified dementia.

#### Covariates

Covariates included known risk factors for a dementia diagnosis, including time-updated calendar period (1997–2004, 2005–2009, 2010–2017, to account for changes in dementia diagnosis and treatment), [14] time since bereavement, socioeconomic status (from IMD data), clinically diagnosed depression (measured >1 year pre-index date, as diagnosis may be affected by upcoming partner death and defined as having a diagnosis or symptom of depression), and the following measured at cohort entry: alcohol use, smoking status, body mass index (BMI, according to WHO categories), hearing loss, hypertension, diabetes, and carer status. Post-bereavement mediators included time-updated depression, time-updated diabetes and consultation rate following bereavement (including all face-to-face consultations for any reason, which may include bereavement and or dementia-related reasons). We did not adjust for consultation frequency (as it may be dependent on dementia status), but instead tabulated yearly consultation rate by exposure.

Full variable definitions can be found in the Supplementary Material.

### Statistical methods

#### Primary analyses

Patients with missing BMI, smoking, and alcohol data were excluded from the final analysis sample as these data are unlikely to be missing at random, making multiple imputation inappropriate [15]. We described the baseline characteristics and crude incidence of dementia in the bereaved and non-bereaved cohorts. Cox regression models stratified on matched pair were used to generate crude and adjusted hazard ratios (HRs) for the association between partner bereavement and diagnosed dementia. There was very strong evidence against the proportional hazards assumption (Schöenfeld residuals test, *p* = 0.001), suggesting the association between time since bereavement and dementia varied over time. Time since bereavement was split into overlapping follow-up periods (0–3 months, 0–6 months, 0–1 years, 0–2 years, 0–5 years, total follow-up) to account for depletion of susceptibles (where over follow-up the proportion of individuals susceptible to dementia increases in the non-bereaved cohort compared with the bereaved cohort, because shortly after bereavement those most “susceptible” to the outcome are removed) [16]. Our minimally adjusted model was stratified by matched set, with current age as the underlying timescale, and adjusted for calendar time, IMD, and time since index date. Our second model additionally adjusted for other possible confounders.

#### Secondary analyses

We examined the association between partner bereavement and diagnosed dementia by sex of the bereaved, age at bereavement (30–64, 65–74, 75–84, ≥85 years), and calendar time period. We examined the association between our secondary exposures (predicted risk of death and living alone during the bereavement period) and dementia. Finally, we added each potential mediator one by one.

#### Sensitivity analyses

A range of sensitivity analyses were conducted to check our assumptions (see Table 1).

**Table 1:** Sensitivity analyses conducted to check our assumptions

Sensitivity analysis	Justification
The primary analysis was repeated excluding patients with memory problems (identified from Read codes) prior to study start.	To reduce the number of patients entering the study with early or undiagnosed dementia.
The primary analysis was repeated restricting study start to 1 April 2005.	Recording dementia diagnoses were incentivised through the Quality and Outcomes Framework, a pay-for-performance scheme^1^, from 2005.
The primary analysis was repeated using the latest of two dementia diagnoses on separate dates within one year.	Requiring two dementia diagnoses is in case the first dementia diagnosis was initially misclassified.
The primary analysis was repeated restricting to patients with linked data only.	To improve the ascertainment of dementia diagnoses, which may not be as fully captured in primary-care records alone.
Predicted risk of death was redefined using terminal disease (identified by primary care and hospital records for terminal disease (as indicated by, e.g., stay at hospice), instead of the Charlson Comorbidity Index.	Charlson comorbidity index was not developed as a tool to predict risk of death, therefore an alternative measure of risk of death was used to substantiate these analyses.
We used an alternative control outcome, acute cardiovascular events (myocardial infarction (MI) and stroke).	To validate the partner bereavement algorithm, as there is a known association between partner bereavement and these outcomes.
In a *post-hoc* sensitivity analysis, we analysed the association between bereavement and dementia diagnosis by country within the UK.	The rate of dementia diagnosis differs substantially by country (reported proportion with dementia who receive a diagnosis: Wales: 53%, Scotland: 67%, Northern Ireland: 73% and England: 70%)^2^ therefore we hypothesised that the association may vary by country.

1. NHS Employers. Quality and outcomes framework. 2. Alzheimer’s Research UK. Dementia statistics hub: Diagnoses in the UK.

#### Data availability statement

CPRD data on which the analysis was based is held securely by the London School of Hygiene and Tropical Medicine, under the CPRD data access license (https://www.cprd.com/dataAccess/).

## RESULTS

After matching (on age and sex) and removing individuals with existing dementia, there were 307,202 patients (153,601 matched exposed/unexposed pairs) (Supplementary Figure 1). Of these, 58,615 (19.1%) were excluded from subsequent analyses due to incomplete data on analysis variables in one or both matched pairs (7.0% missing BMI, 1.5% missing smoking, 7.3% missing alcohol status, 8.9% no remaining matched pair). This left 247,586 people in the final analysis sample. The distributions of variables did not differ greatly between those selected in the final analysis sample (N = 247,586) and the overall study sample (N = 307,202) (see Supplementary Material, Supplementary Table 1).

The median age of the final analysis sample was 74.2 years (IQR:66.9–80.4) at study entry (Table 2). Those experiencing partner bereavement were more likely to be deprived (23.2% of bereaved versus 20.1% of non-bereaved in the most deprived quintile), current smokers (16.0% versus 11.0%), have depression (24.5% versus 22.2%), and diabetes (10.4% versus 9.0%). Median follow-up was higher in those bereaved (4.4 years, IQR: 1.9–8.0) than non-bereaved (4.0 years, IQR:1.7–7.4), as those non-bereaved had some additional censoring (specifically, they may have become bereaved during follow-up or their partner may have left the practice).

**Table 2:** Baseline and demographic characteristics by partner bereavement exposure status. Figures are numbers (percentage) unless otherwise stated

	Partner bereavement *n* (%)	No partner bereavement *n* (%)
No of patients	123793 (100%)	123793 (100%)
CPRD follow-up prior to index date, median (IQR)	9.8 (6.0–14.0)	9.4 (5.6–13.7)
CPRD follow-up post-index date, median (IQR)	4.4 (1.9–8.0)	4.0 (1.7–7.4)
Females	81336 (65.7)	81336 (65.7)
Age in years (median, IQR)	74.2 (66.9–80.4)	74.2 (66.9–80.4)
Age (years)
30–54	5681 (4.6)	5681 (4.6)
55–64	19406 (15.7)	19406 (15.7)
65–75	40434 (32.7)	40434 (32.7)
75–84	45536 (36.8)	45536 (36.8)
≥85	12736 (10.3)	12736 (10.3)
Socioeconomic status (practice-level)*
1 (least deprived)	23366 (18.9)	26265 (21.2)
2	21144 (17.1)	22517 (18.2)
3	24207 (19.6)	25041 (20.2)
4	26306 (21.2)	25119 (20.3)
5 (most deprived)	28770 (23.2)	24851 (20.1)
Health and lifestyle factors
Carer	5952 (4.8)	1938 (1.6)
Marital discord	1332 (1.1)	1227 (1.0)
BMI category
Underweight	3028 (2.4)	2276 (1.8)
Normal Weight	44690 (36.1)	45182 (36.5)
Overweight	46941 (37.9)	49005 (39.6)
Obese	29134 (23.5)	27330 (22.1)
Smoking status
non-smoker	54728 (44.2)	60836 (49.1)
current smoker	19838 (16.0)	13669 (11.0)
ex-smoker	49227 (39.8)	49288 (39.8)
Alcohol use
Non-drinker	14725 (11.9)	13361 (10.8)
Current drinker	91212 (73.7)	95118 (76.8)
Ex-drinker	17856 (14.4)	15314 (12.4)
Medical conditions at baseline
Depression†	30332 (24.5)	27500 (22.2)
Diabetes	12933 (10.4)	11100 (9.0)
Hypertension	57694 (46.6)	56504 (45.6)
Hearing loss	20656 (16.7)	20873 (16.9)

CPRD, Clinical Practice Research Datalink; BMI, body mass index. ^*^Measured by Index of Multiple deprivation score †Measured > 1 year prior to study entry.

In total, 5,697 (4.6%) of non-bereaved patients and 6,520 (5.3%) of bereaved patients were diagnosed with dementia during follow-up (Table 3). We first assessed the association by discrete time periods after bereavement (Supplementary Table 7), but the results suggested a built-in selection bias, known as ‘depletion of susceptibles’. To reduce this possible bias, we assessed the association by overlapping time periods; we observed an almost 50% increased risk of diagnosed dementia within three months following bereavement (HR 1.43, 95% CI:1.20–1.71), after adjusting for all covariates. By contrast, there was little evidence of an increased risk of diagnosed dementia over the longer-term (HR 0.94, 95% CI:0.89–0.98 within the first 5 years).

**Table 3:** Crude rate for dementia diagnosis by exposure to partner bereavement, and unadjusted and adjusted hazard ratios, by time since partner bereavement

	No of events	Total person time (person-years)	Crude rate (per 1000 person-years)	Crude HR* (95% CI)	Minimally-adjusted HR** (95% CI)	Adjusted HR† (95% CI)
**Time since bereavement**
*0*–*3 months*
No partner bereavement	254	30337	8.37 (7.40–9.47)	1.00	1.00	1.00
Partner bereavement	367	30288	12.12 (10.94–13.42)	1.45 (1.23–1.70)	1.46 (1.24–1.72)	1.43 (1.20–1.71)
*0*–*6 months*
No partner bereavement	497	59462	8.36 (7.65–9.13)	1.00	1.00	1.00
Partner bereavement	626	59353	10.55 (9.75–11.41)	1.26 (1.12–1.42)	1.26 (1.12–1.43)	1.24 (1.09–1.41)
*0*–*12 months*
No partner bereavement	948	114201	8.30 (7.79–8.85)	1.00	1.00	1.00
Partner bereavement	1055	114197	9.24 (8.70–9.81)	1.11 (1.02–1.21)	1.09 (0.99–1.20)	1.07 (0.97–1.18)
*0*–*2 years*
No partner bereavement	1718	210785	8.15 (7.77–8.55)	1.00	1.00	1.00
Partner bereavement	1800	212233	8.48 (8.10–8.88)	1.04 (0.97–1.11)	1.02 (0.95–1.09)	0.99 (0.92–1.07)
*0*–*5 years*
No partner bereavement	3549	416015	8.53 (8.25–8.82)	1.00	1.00	1.00
Partner bereavement	3676	428840	8.57 (8.30–8.85)	1.00 (0.96–1.05)	0.95 (0.90–1.00)	0.92 (0.87–0.97)
Complete follow-up
No partner bereavement	5697	613802	9.28 (9.04–9.53)	1.00	1.00	1.00
Partner bereavement	6520	658900	9.90 (9.66–10.14)	1.06 (1.02–1.09)	0.96 (0.91–1.01)	0.94 (0.89–0.98)

HR, hazard ratio; CI, confidence interval. *Cox model with time in study time scale, and no stratification by matched set. **Cox model with age time scale, stratified by matched set, additionally adjusted for sex, calendar time, IMD and time since bereavement. †Additional adjustment for: BMI, smoking status, alcohol use, depression, diabetes, hearing loss, hypertension and carer status.

**Fig.1:**
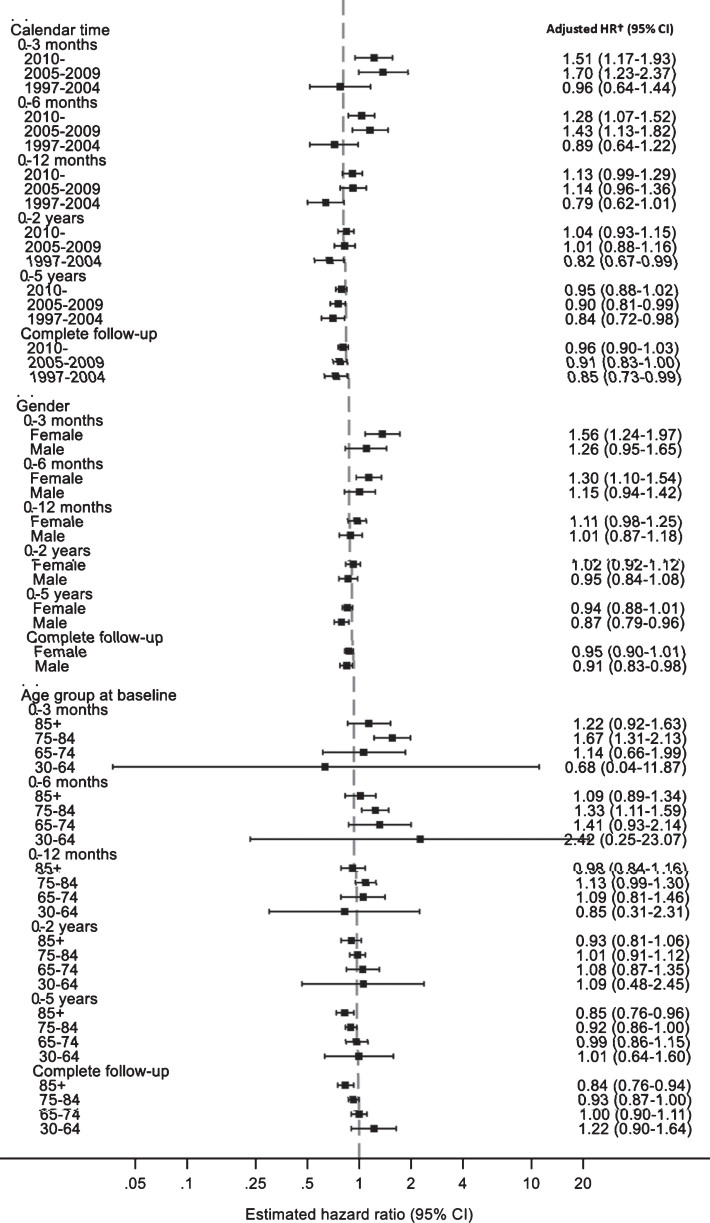
Adjusted hazard ratios for the association between partner bereavement and diagnosed dementia, by age and calendar time at bereavement and gender of bereaved, stratified by time since bereavement. HR, hazard ratio; CI, confidence interval. ^†^Cox model with age timescale, stratified by matched set, additionally adjusted for sex, calendar time, Index of Multiple Deprivation, time since bereavement, body mass index, smoking status, alcohol use, depression, diabetes, hearing loss, hypertension, and carer status.

We examined whether age, gender, and calendar period modified the association between partner bereavement and diagnosed dementia, by certain characteristics (Fig. 1 and Supplementary Table 2). There was no evidence of effect modification by sex or age of the bereaved; however, the confidence intervals were very wide for the youngest age group (30–64 years) due to the small number of participants in this age group. Evidence of a short-term increased risk of dementia diagnosis was only observed for those followed-up after 2004. In *post-hoc* analyses, we found the magnitude of association between bereavement and diagnosed dementia (within three months) by country correlated with the proportions of undiagnosed dementia (Supplementary Figure 2); the association was greatest in Wales (HR 2.84, 95% CI:1.23–6.56).

When assessing our secondary exposures, there was some evidence of a dose-response relationship between predicted risk of partner death and diagnosis of dementia in the first 3-months post-bereavement (Supplementary Figure 3): individuals whose partner was at high risk of death were at greatest risk post-bereavement (however, the confidence intervals overlapped).

Adjusting for the mediators (time-updated depression and time-updated diabetes) did not alter the results (Supplementary Table 3). Those bereaved had a higher consultation rate within 3 months of bereavement (16.0 versus 12.0 per year) and this remained elevated over the complete follow-up period (15.9 versus 14.4 per year).

### Sensitivity analyses

Excluding patients with prior recorded memory problems (*n* = 14,642 (6.0%) dropped) did not alter the results (Supplementary Table 4). After starting follow-up at 1 April 2005 (*n* = 232,516) and restricting to patients with linked data only (*n* = 89,926), the magnitude of association between bereavement and diagnosed dementia within three months increased (Supplementary Table 4). After requiring two dementia diagnoses to define dementia, the point estimates followed the same pattern, though were greatly attenuated and precision was reduced. In the alternative outcome analyses, partner bereavement was associated with acute cardiovascular events within three months (HR 1.27, 95% CI:1.06–1.53), which reduced over time since bereavement (Supplementary Table 5). Finally, the magnitude of association between bereavement and diagnosed dementia within three months was larger for those whose partner had died from a terminal/end-stage illness (compared to non-terminal illness) (HR 2.10, 95% CI:1.08–4.06) (Supplementary Table 6) and the association was similar among those living alone (HR 1.42 95% CI: 1.16–1.74) and not living alone (HR 1.47, 95% CI 1.04–2.07) during the period of bereavement.

## DISCUSSION

### Principal findings

In this large, UK population-based cohort study of over 200,000 individuals, partner-loss appeared to trigger the detection of dementia; bereavement was associated with a 43% increased risk of receiving a dementia diagnosis within three months, a 24% increased risk within six months, and no evidence of an increased risk over longer time periods. We hypothesized that the immediate increased risk of a dementia diagnosis may be from existing undiagnosed dementia becoming apparent on partner death. Due to the long pre-clinical phase of dementia, it is unlikely that the short-term increased risk is a direct causal effect of the bereavement. Over the longer term, it appears that dementia is under-ascertained in UK health records among those who have lost their partner. Greater awareness of the time following bereavement as a high-risk period for dementia diagnosis, as well as the apparent under ascertainment of dementia in those widowed for some years, could speed up diagnoses and allow patients to receive necessary support.

### Strengths and weaknesses

This study has several strengths. First, it is one of the largest studies to explore whether partner bereavement is a risk factor for receiving a dementia diagnosis. Second, it is, to our knowledge, the first study to investigate dementia diagnoses in a range of time windows following a partner bereavement, including immediately following bereavement. Finally, by using CPRD data, we were able to adjust for important potential confounders, such as smoking and BMI, unavailable in some previous studies [17].

The study also has some limitations. First, type of dementia was unavailable; these data may further our understanding of the mechanisms linking bereavement and dementia. Second, dementia records in some bereaved patients may be misdiagnosed, perhaps due to misdiagnosed depression (induced by bereavement) or partner loss causing bereaved individuals to underperform in cognitive tests. However, the positive predictive value (PPV) of a dementia diagnosis in CPRD is around 80–90% and the diagnosis of dementia is fairly rigorous, typically formulated within specialist memory clinics, making misdiagnosis unlikely [18, 19]. We have also adjusted for depression; therefore we do not believe the association is driven by late-life depression. Finally, our study relies on an in-house developed algorithm to identify partners; although the algorithm is limited (for example, it cannot identify persons who remarry), we observed the known increased risk of acute cardiovascular events following bereavement, suggesting it is sufficiently reliable.

### Comparison previous literature and mechanisms

Previous research indicates that widowed individuals are at greater risk of dementia compared to their married counterparts [5], with evidence coming from seven longitudinal studies where dementia was systematically ascertained at baseline and during follow-up (ranging from a mean of 3 to 20 years) by clinicians [20]. These studies differ from ours as they are investigating whether partner bereavement is a causal risk factor for dementia, rather than whether partner bereavement affects the risk of receiving a dementia diagnosis. However, one major problem with these “causal” studies is the short follow-up, as dementia has a long pre-clinical period during which cognitive decline occurs; only one of the seven studies [21] had a mean follow-up of over 10 years, and this study found no association between partner loss and dementia.

Our study utilized existing medical records, in which dementia is known to be under-captured; studies suggest around one third of individuals in UK-settings with dementia are undiagnosed [22, 23]. The initial increased risk of receiving a dementia diagnosis in the six months following bereavement could therefore reflect undiagnosed dementia being recognized, as a result of bereavement. First, the deceased partner may have been compensating for decline in functioning due to dementia; upon their death, the surviving partner may not cope with activities of daily living, thus uncovering dementia. Second, prior to and following partner bereavement, contact with healthcare services may increase, leading to greater opportunity for a dementia diagnosis around this time period; the association was greatest in Wales (HR 2.84, 95% CI:1.23–6.56) where there is a higher rate of undiagnosed dementia (only 53% of patients with dementia are reported to receive a diagnosis in Wales [1]). Third, family and friends often gather together in the immediate aftermath of a bereavement to support the surviving partner, perhaps facilitating a dementia diagnosis.

It is very unlikely that partner bereavement is directly causing the increased risk of dementia within 6 months following bereavement. Bereavement is a very acute stressor, which could cause a direct assault on cognition or function for people vulnerable to dementia, who have less resilience to age-related neuropathology. However, dementia is a progressive disorder, with a long pre-clinical phase hypothesized to begin decades prior to diagnosis; therefore, the effects of bereavement are unlikely to manifest so quickly on dementia risk.

We observed no increased risk of receiving a diagnosis of dementia over complete follow-up. Many rigorous longitudinal studies with good capture of dementia and several years of follow-up have reported that widowed individuals are more likely to develop dementia [5]. The long pre-clinical phase of dementia may mean our study lacked sufficient follow-up to detect the long-term effect of bereavement on cognitive decline. However, what our study uniquely suggests is that widowed individuals may be more vulnerable to their dementia going undetected; US data suggests that older, unmarried individuals are less likely to be diagnosed in routine practice [24] and qualitative research from the UK indicates diagnosing dementia in those attending clinic alone is difficult [25]. A dementia diagnosis requires evidence of progressive cognitive decline, which clinicians gather from their own observations and from friends and family, often referred to as ‘collateral history’. Without a partner to verify an individual’s symptoms of cognitive decline, dementia diagnosis may be overlooked.

Our findings differ from a Swedish cohort study using routinely collected healthcare data which suggested that dementia risk was greater in the bereaved compared to married individuals over three years of follow-up [17]. Under-diagnosis of dementia is similarly high in these Swedish registries as it is in UK medical records [26]. However, the design of the Swedish study had some key differences. Time since bereavement was not assessed; instead, participants entered as either married, single, divorced, or widowed so it is not clear how long individuals had been widowed. Additionally, dementia was derived from hospitalization and death data only (including both primary and secondary diagnoses); bereaved individuals could present to hospital more than their married counterparts, leading to earlier dementia diagnoses.

### Unanswered questions and future research

This study highlights the usefulness of routinely collected medical records for investigating risk factors for receiving a dementia diagnosis in clinical care. Greater exploration of the patterns and causes of under-diagnosis, within different contexts, may be helpful for future epidemiological studies investigating dementia, as well as for clinicians and policy decision-makers aiming to increase the proportion of dementia patients with a formal dementia diagnosis [27]. Better data on the partner relationship and other social support networks may provide more detail on how the effect of bereavement on dementia diagnosis may be modified by these social factors.

### Conclusions

This study found that bereavement triggers a short-term increased risk of receiving a diagnosis of dementia; within the three months following partner bereavement there was a 47% increased risk of the surviving partner receiving a diagnosis of dementia. We hypothesized that bereavement acts as a trigger for recognizing existing undiagnosed dementia in the months following partner-loss. This increased risk of being diagnosed with dementia reduced over longer time periods, with no increase observed over time periods of 12 months or longer from bereavement. With rigorous follow-up studies indicating that being a widow/widower increases the risk of dementia, this study suggests that over the longer-term, widows/widowers are experiencing greater under-diagnosis of dementia in routine clinical care compared to their partnered counterparts. Greater awareness of the time around bereavement being a high-risk period for dementia diagnosis may help facilitate dementia diagnoses and allow individuals to gain appropriate support.

## Supplementary Material

Supplementary Material

Supplementary Tables
