# Supplementary material for: Partner Bereavement and Detection of Dementia: A UK-Based Cohort Study Using Routine Health Data
Source: J Alzheimers Dis. 2019 Nov 12;72(2):653–62. doi: 10.3233/JAD-190571 (PMC6918907; doi:10.3233/JAD-190571)
Supplement: Supplementary Material [file jad-72-jad190571-s001.docx]

**Supplementary Material**

**Partner Bereavement and Detection of Dementia: A UK-Based Cohort Study Using Routine Health Data**

**±†Partner algorithm**

“The algorithm to identify partners is based on the family number in the CPRD. This number identifies people in a practice who are living in the same household or otherwise are associated. First, we will identify the entire CPRD population with acceptable standard. For each practice, we will then identify the earliest available date in the study inclusion period (1 January 1997 to 31 July 2016 [or later depending on availability at time of study initiation]) for each practice. This date will be the ‘study initiation date’. We will identify the household composition (i.e., family numbers registered) and restrict to persons who are alive and registered with the practice on that study initiation date. We exclude persons who are not linked to any other patient through their family practice number (assumed single). Also, to avoid misclassification based on inconsistent use of the family number (e.g., for persons living in the same flat block or nursing home), we will not consider cohabitees to be potential partners where the family number is used for >10 persons. For each family number, we will then identify couples of the opposite sex, with an age gap of ≤10 years and with no younger adult in the household within 15 years of either of the couple. We will exclude couples where both individuals are <40 years, based on the assumption that cohabitation between friends (e.g., in a flat or student dorm) and within a family is more common at younger age. Similarly, to avoid including otherwise-associated persons (e.g., nursing home residents), we will exclude couples where both individuals are 95 years or older or where a person in the couple has any code indicating residence in a communal establishment before the study initiation date.

The method of identifying partners has been developed by adapting an algorithm previously applied on THIN data with great success in linking partner bereavement with increased risk of death, medical care, and cardiovascular disease [1-4]. One of the studies reported that according to national representative household surveys in England, 99% of cohabitating couples aged ≥60 years who are of the opposite sex and have an age difference of <10 years identify themselves as partners [1]. Furthermore, we have previously applied the algorithm to study the association between partner bereavement and herpes zoster using a case-control design, finding results comparable to a study from the U.S. and Denmark [5, 6].

We will follow identified couples from the study initiation date to the last collection date, transfer out of practice, or end of study. If one or both persons of a couple transfer out of the practice, both will be censored from that date. Death of one of the members of the couple during this period will then be identified and the bereaved (surviving) partner will be included in the bereaved/exposed cohort. The date of partner death will be considered the index date for the study. We will require at least 1 year of standard follow-up prior to this index date.”

**Variable definitions**

*Manner of partner death*

We calculated an age-adjusted Charlson Comorbidity Index (CCI) score at one month before death for partners of the exposed cohort. The CCI assigns 0 to 6 points to a range of chronic diseases according to their ability to predict death, with additional points given according to age [7]. Based on the total score, the risk of partner death was classified as low (0­–3 points), intermediate (4–6 points) or high (≥7 points).

*Terminal illness*

Terminal illness codes were identified in primary and secondary care records.

*Smoking status*

Smoking was defined at index date using three categories; non-smoker, current smoker and ex-smoker. Data were derived from: 1) medical Read codes such as “tobacco dependence in remission” and “stopped smoking”; and 2) data from structured data fields in the additional details file, specifically entity type number four records information on smoking, including the patients smoking “status” (yes, no or ex) and the “number of cigarettes per day” smoked. These data were extracted for each patient along with the date the status was assigned.

Smoking status was then classified by the algorithm shown in Diagram 1.

**Diagram 1. Algorithm used to define BMI, smoking, and alcohol status**

Note: the nearest record to the index date from the first choice period was taken if available, otherwise from the second choice period and so on. If patients had any evidence of smoking or alcohol prior to index date, but were defined by the algorithm as a non-smoker or non-drinkers, they were reclassified as ex-smokers or ex-drinkers.

Fourth choice

Third choice

Index date

1 year before index date

1 month after index date

1 year after index date

First choice

Second choice

Third choiceThird choice


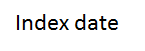


Fourth choice

*BMI category*

*BMI* is a metric used to classify people as under- or overweight. It is derived from patients weight in kilograms / (height in meters)². In this study BMI was defined at index date, according to adult BMI cut-offs defined by the WHO, as underweight (BMI <18.5), healthy weight (BMI 18.5-24.9), overweight (BMI 25+), or obese (BMI ≥30).

Height and weight are recorded opportunistically in UK primary care and are contained in the CPRD additional details files, in entity types 104 and 140. Data cleaning was based on that described in the paper by Bhaskaran et al. on BMI recordings in CPRD [8]. Any records taken when the patient was <16 years old were dropped, as the patient would still be growing. Implausible values were excluded, specifically height records <4 and >7 foot were dropped, and weight records <20 kilograms were dropped. For each weight record, a BMI was calculated using first, height recorded on that day, otherwise, last observation of height, or if no previous height records were available, then first future height.

BMI status was then classified by the algorithm shown in Diagram 1.

Read codes exist classifying patients by BMI category, however they are very rarely used (around 3% of patients have a clinical code that would enable classification into WHO BMI categories) [8], therefore Read codes were not incorporated into the definition of BMI.

*Alcohol use*

Alcohol use was defined at index date and classified as non-drinker, ex-drinker, or current drinker using: 1) medical Read codes, such as “alcohol problem drinking”; and 2) data from structured data fields in the additional details file, specifically entity type 5 records data on alcohol use, including the patients alcohol “status” (yes, no or ex) and the number of alcohol “units per week” consumed.

Alcohol status was then classified by the algorithm shown in Diagram 1.

**REFERENCES**

[1] Shah SM, Carey IM, Harris T, DeWilde S, Victor CR, Cook DG (2012) Do good health and material circumstances protect older people from the increased risk of death after bereavement? *Am J Epidemiol* **176**, 689-698.

[2] Shah SM, Carey IM, Harris T, DeWilde S, Victor CR, Cook DG (2013) The effect of unexpected bereavement on mortality in older couples. *Am J Public Health* **103**, 1140-1145.

[3] Carey IM, Shah SM, DeWilde S, Harris T, Victor CR, Cook DG (2014) Increased risk of acute cardiovascular events after partner bereavement: a matched cohort study. *JAMA Intern Med* **174**, 598-605.

[4] King M, Vasanthan M, Petersen I, Jones L, Marston L, Nazareth I (2013) Mortality and medical care after bereavement: a general practice cohort study. *Plos One* **8**, e52561.

[5] Harpaz R, Leung JW, Brown CJ, Zhou FJ (2015) Psychological stress as a trigger for herpes zoster: might the conventional wisdom be wrong? *Clin Infect Dis* **60**, 781-785.

[6] Schmidt SAJ, Vestergaard M, Pedersen HS, Schonheyder HC, Thomas SL, Smeeth L, Mansfield KE, Sorensen HT, Forbes HJ, Langan SM (2017) Partner bereavement and risk of herpes zoster: results from two population-based case-control studies in Denmark and the United Kingdom. *Clin Infect Dis* **64**, 572-579.

[7] Charlson M, Szatrowski TP, Peterson J, Gold J (1994) Validation of a combined comorbidity index. *J Clin Epidemiol* **47**, 1245-1251.

[8] Bhaskaran K, Forbes HJ, Douglas I, Leon DA, Smeeth L (2013) Representativeness and optimal use of body mass index (BMI) in the UK Clinical Practice Research Datalink (CPRD). *BMJ Open* **3**, e003389.

**Supplementary Figure 1. Flow diagram of study participants**

**
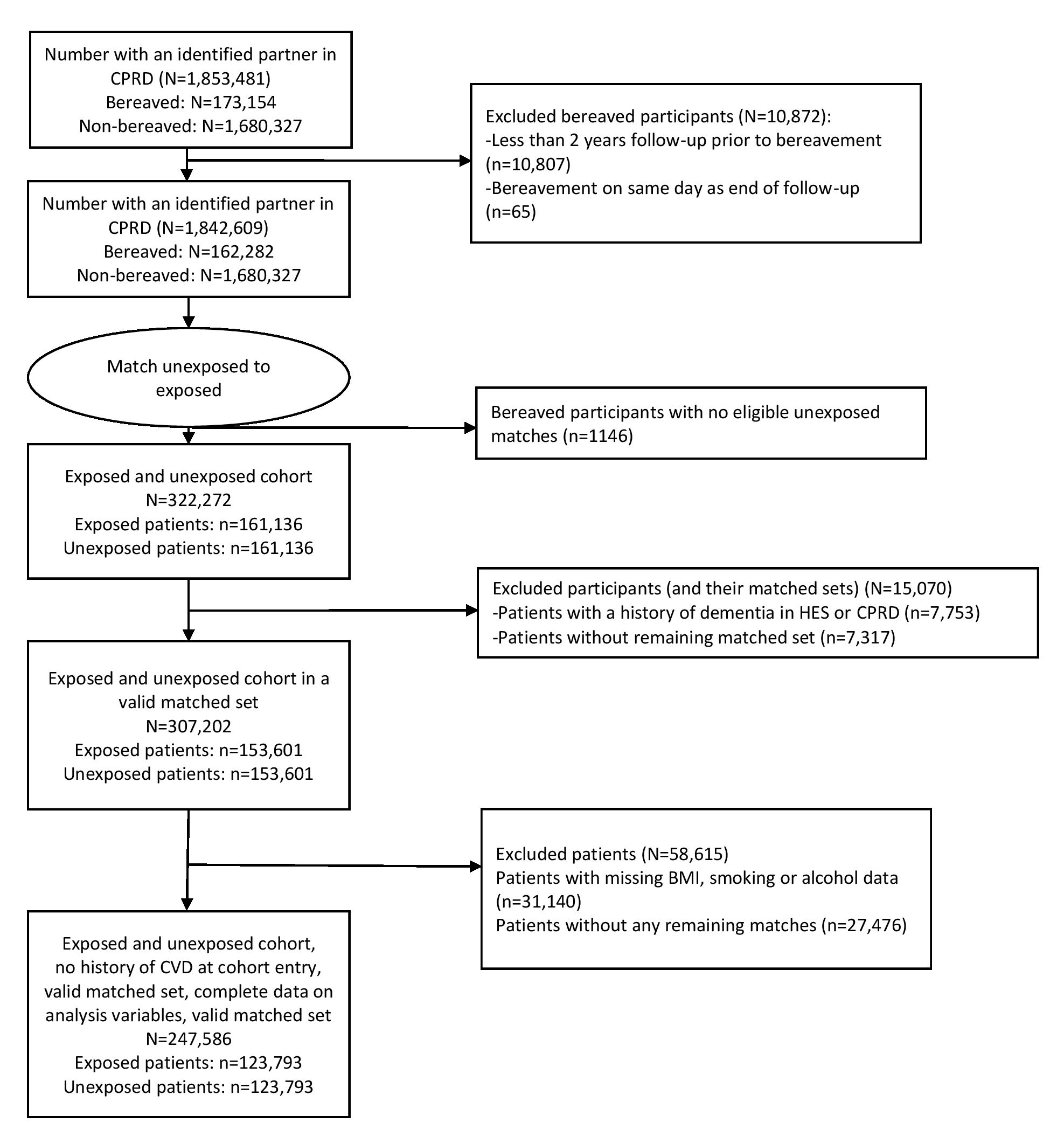
**

**Supplementary Figure 2.** Adjusted hazard ratios for the association between partner bereavement and diagnosed dementia, by country in the UK and time since bereavement (Proportion with dementia who receive a dementia diagnosis in; Wales-53%, Scotland-67%, Northern Ireland-73%, England-70%).


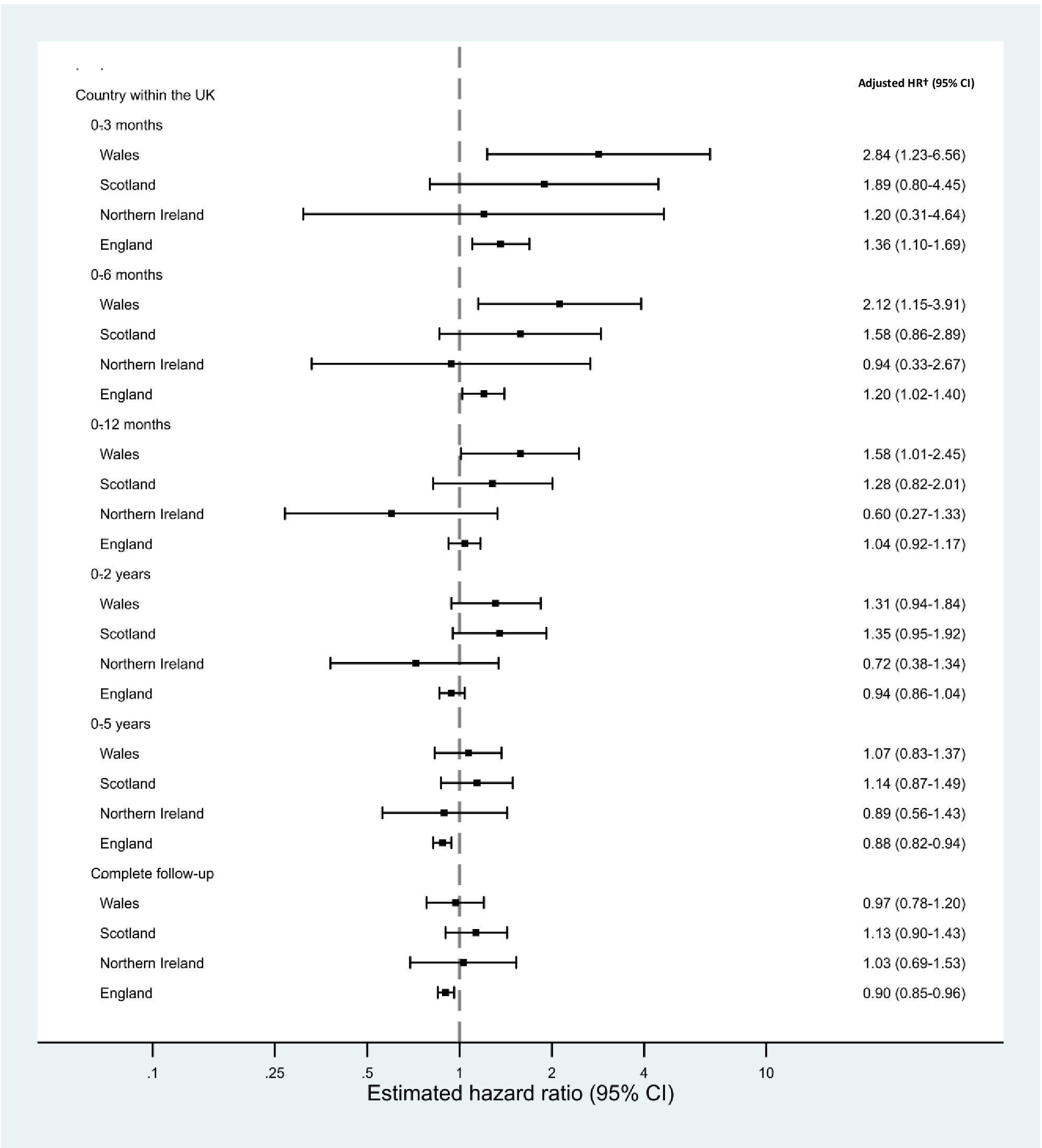


HR, hazard ratio; CI, confidence interval. ^†^Cox model with age timescale, stratified by matched set, additionally adjusted for sex, calendar time, IMD, time since bereavement, BMI, smoking status, alcohol use, depression, diabetes, hearing loss, hypertension, and carer status.

**Supplementary Figure 3.** Adjusted hazard ratios for the association between our secondary exposures (predicted risk of death and living alone at bereavement) and dementia stratified by time since bereavement.


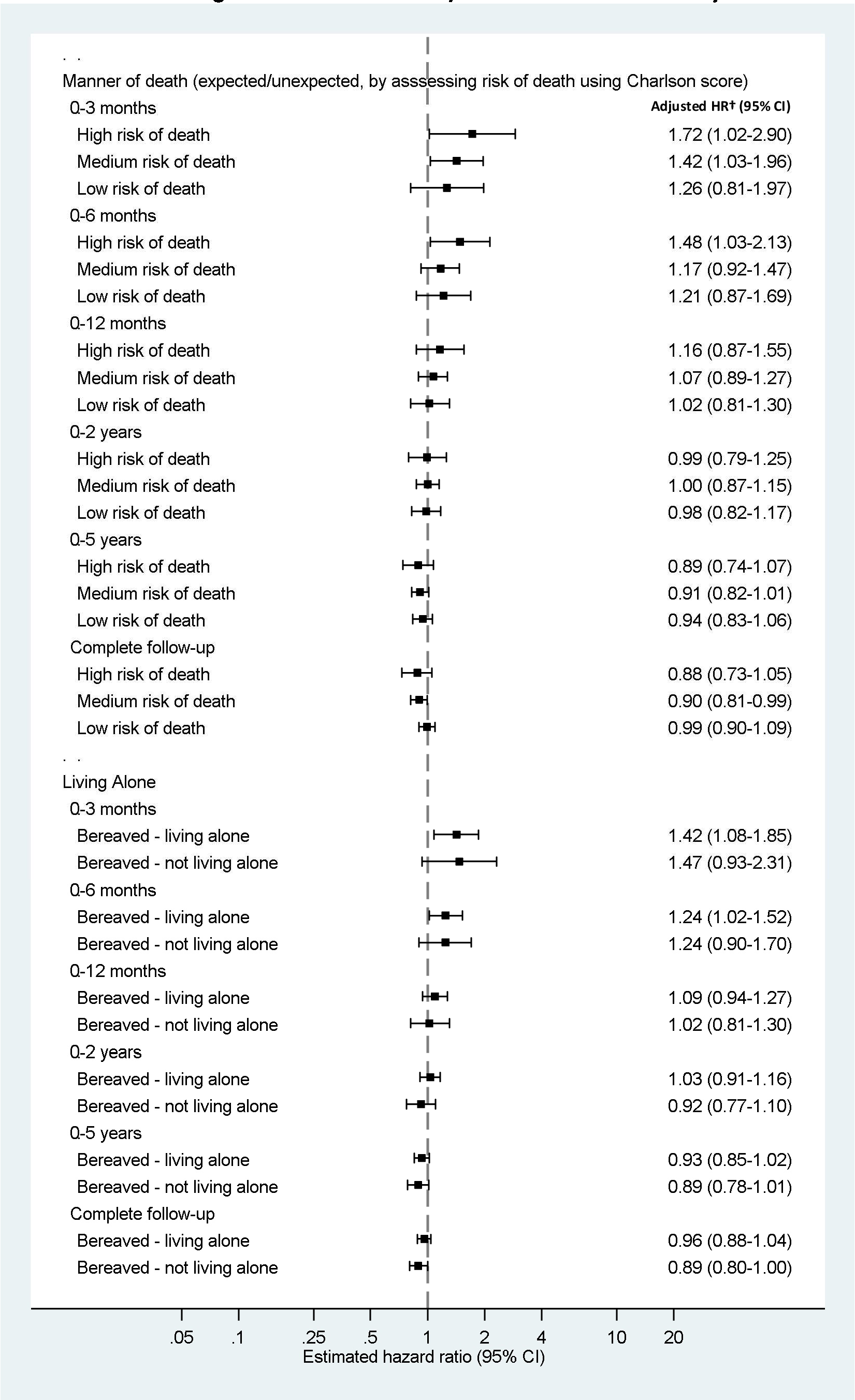


HR, hazard ratio; CI, confidence interval. ^†^Cox model with age timescale, stratified by matched set, additionally adjusted for sex, calendar time, Index of Multiple Deprivation, time since bereavement, body mass index, smoking status, alcohol use, depression, diabetes, hearing loss, hypertension, and carer status.
