# Supplementary material for: Partner Bereavement and Detection of Dementia: A UK-Based Cohort Study Using Routine Health Data
Source: J Alzheimers Dis. 2019 Nov 12;72(2):653–62. doi: 10.3233/JAD-190571 (PMC6918907; doi:10.3233/JAD-190571)
Supplement: Supplementary Tables [file jad-72-jad190571-s002.docx]

| **Supplementary Table 1. Comparison of baseline and demographic characteristics by partner bereavement exposure status, for those included in the final sample and those in the overall sample. Figures are numbers (percentage) unless otherwise stated** | | | | |  |  |  |
| --- | --- | --- | --- | --- | --- | --- | --- |
|  | **Final analysis sample:** those with complete data on all variables (N=247,586) | | **Overall sample:** including those with missing BMI, smoking and alcohol status (N=307,202) | |  |  |  |
|  | Partner bereavement n (%) | No partner bereavement n (%) | Partner bereavement n (%) | No partner bereavement n (%) |  |  |  |
| No of patients | 123793 (100%) | 123793 (100%) | 153601 (100%) | 153601 (100%) |  |  |  |
| CPRD follow-up prior to index date, median (IQR) | 9.8 (6.0-14.0) | 9.4 (5.6-13.7) | 9.5 (5.8-13.7) | 9.1 (5.4-13.3) |  |  |  |
| CPRD follow-up post-index date, median (IQR) | 4.4 (1.9- 8.0) | 4.0 (1.7- 7.4) | 4.3 (1.8- 7.9) | 3.9 (1.7- 7.2) |  |  |  |
| Females | 81336 (65.7) | 81336 (65.7) | 100432 (65.4) | 100432 (65.4) |  |  |  |
| Age (years) |  |  |  |  |  |  |  |
| 30-54 | 5681 ( 4.6) | 5681 ( 4.6) | 7242 ( 4.7) | 7242 ( 4.7) |  |  |  |
| 55-64 | 19406 (15.7) | 19406 (15.7) | 23364 (15.2) | 23364 (15.2) |  |  |  |
| 65-75 | 40434 (32.7) | 40434 (32.7) | 47851 (31.2) | 47851 (31.2) |  |  |  |
| 75-84 | 45536 (36.8) | 45536 (36.8) | 56945 (37.1) | 56945 (37.1) |  |  |  |
| ≥85 | 12736 (10.3) | 12736 (10.3) | 18199 (11.8) | 18199 (11.8) |  |  |  |
| Socioeconomic status (practice-level)* |  |  |  |  |  |  |  |
| 1 (least deprived) | 23366 (18.9) | 26265 (21.2) | 29240 (19.0) | 32681 (21.3) |  |  |  |
| 2 | 21144 (17.1) | 22517 (18.2) | 26322 (17.1) | 27882 (18.2) |  |  |  |
| 3 | 24207 (19.6) | 25041 (20.2) | 30214 (19.7) | 31233 (20.3) |  |  |  |
| 4 | 26306 (21.2) | 25119 (20.3) | 32592 (21.2) | 31215 (20.3) |  |  |  |
| 5 (most deprived) | 28770 (23.2) | 24851 (20.1) | 35233 (22.9) | 30590 (19.9) |  |  |  |
| **Health and lifestyle factors** |  |  |  |  |  |  |  |
| Carer | 5952 ( 4.8) | 1938 ( 1.6) | 6886 ( 4.5) | 2262 ( 1.5) |  |  |  |
| Marital discord | 1332 ( 1.1) | 1227 ( 1.0) | 1595 ( 1.0) | 1420 ( 0.9) |  |  |  |
| BMI category |  |  |  |  |  |  |  |
| *Underweight* | 3028 ( 2.4) | 2276 ( 1.8) | 3630 ( 2.4) | 2762 ( 1.8) |  |  |  |
| *Normal Weight* | 44690 (36.1) | 45182 (36.5) | 51921 (33.8) | 52937 (34.5) |  |  |  |
| *Overweight* | 46941 (37.9) | 49005 (39.6) | 53755 (35.0) | 56494 (36.8) |  |  |  |
| *Obese* | 29134 (23.5) | 27330 (22.1) | 33021 (21.5) | 31206 (20.3) |  |  |  |
| *Missing* |  |  | 11274 ( 7.3) | 10202 ( 6.6) |  |  |  |
| Smoking status |  |  |  |  |  |  |  |
| *non-smoker* | 54728 (44.2) | 60836 (49.1) | 68630 (44.7) | 76268 (49.7) |  |  |  |
| *current smoker* | 19838 (16.0) | 13669 (11.0) | 24674 (16.1) | 17061 (11.1) |  |  |  |
| *ex-smoker* | 49227 (39.8) | 49288 (39.8) | 57969 (37.7) | 58141 (37.9) |  |  |  |
| *Missing* |  |  | 2328 ( 1.5) | 2131 ( 1.4) |  |  |  |
| Alcohol use |  |  |  |  |  |  |  |
| *non* | 14725 (11.9) | 13361 (10.8) | 17629 (11.5) | 16192 (10.5) |  |  |  |
| *curr* | 91212 (73.7) | 95118 (76.8) | 104478 (68.0) | 109252 (71.1) |  |  |  |
| *ex* | 17856 (14.4) | 15314 (12.4) | 19950 (13.0) | 17304 (11.3) |  |  |  |
| *Missing* |  |  | 11544 ( 7.5) | 10853 ( 7.1) |  |  |  |
| **Medical conditions at baseline** | | | |  |  |  |  |
| Depression^†^ | 30332 (24.5) | 27500 (22.2) | 36174 (23.6) | 32863 (21.4) |  |  |  |
| Diabetes | 12933 (10.4) | 11100 ( 9.0) | 14755 ( 9.6) | 12806 ( 8.3) |  |  |  |
| Hypertension | 57694 (46.6) | 56504 (45.6) | 68159 (44.4) | 67096 (43.7) |  |  |  |
| Hearing loss | 20656 (16.7) | 20873 (16.9) | 24911 (16.2) | 25158 (16.4) |  |  |  |
| *Measured by Index of Multiple deprivation score | |  |  |  |  |  |  |
| ^†^Measured >1 year prior to study entry | |  |  |  |  |  |  |
|  |  |  |  |  |  |  |  |
|  |  |  |  |  |  |  |  |
|  |  |  |  |  |  |  |  |
|  |  |  |  |  |  |  |  |
|  |  |  |  |  |  |  |  |
|  |  |  |  |  |  |  |  |
|  |  |  |  |  |  |  |  |
|  |  |  |  |  |  |  |  |
|  |  |  |  |  |  |  |  |
|  |  |  |  |  |  |  |  |
|  |  |  |  |  |  |  |  |
|  |  |  |  |  |  |  |  |
|  |  |  |  |  |  |  |  |
|  |  |  |  |  |  |  |  |
|  |  |  |  |  |  |  |  |
|  |  |  |  |  |  |  |  |
|  |  |  |  |  |  |  |  |
|  |  |  |  |  |  |  |  |
|  |  |  |  |  |  |  |  |
|  |  |  |  |  |  |  |  |
|  |  |  |  |  |  |  |  |

| **Supplementary Table 2. Crude rate for dementia diagnosis by exposure to partner bereavement, and unadjusted and adjusted hazard ratios, by gender, age at bereavement, calendar year, marital discord at baseline, and manner of death of deceased partner.** | | | | | | | | |  | |  | |  | |  | |  | |  | |  | |  | |  | |  |
| --- | --- | --- | --- | --- | --- | --- | --- | --- | --- | --- | --- | --- | --- | --- | --- | --- | --- | --- | --- | --- | --- | --- | --- | --- | --- | --- | --- |
|  |  | **Number of events** | **Total person time (person-years)** | **Crude rate (per 1000 person-years)** | **Crude HR* (95% CI)** | **Minimally-adjusted HR** (95% CI)** | | **Adjusted HR^†^ (95% CI)** | | **p from Likelihood ratio test** | |  | |  | |  | |  | |  | |  | |  | |  | |
| **Gender** |  |  |  |  |  |  | |  | |  | |  | |  | |  | |  | |  | |  | |  | |  | |
| 0-3 months |  |  |  |  |  |  | |  | |  | |  | |  | |  | |  | |  | |  | |  | |  | |
| Male | No partner bereavement | 108 | 10388.3 | 10.40 (8.61-12.55) | 1.00 | 1.00 | | 1.00 | | 0.23 | |  | |  | |  | |  | |  | |  | |  | |  | |
|  | Partner bereavement | 136 | 10352.5 | 13.14 (11.10-15.54) | 1.26 (0.98-1.63) | 1.32 (1.02-1.72) | | 1.26 (0.95-1.65) | |  |  |  | |  | |  | |  | |  | |  | |  | |  | |
| Female | No partner bereavement | 146 | 19948.7 | 7.32 (6.22-8.61) | 1.00 | 1.00 | | 1.00 | |  |  |  | |  | |  | |  | |  | |  | |  | |  | |
|  | Partner bereavement | 231 | 19935.4 | 11.59 (10.19-13.18) | 1.58 (1.29-1.95) | 1.56 (1.26-1.93) | | 1.56 (1.24-1.97) | |  |  |  | |  | |  | |  | |  | |  | |  | |  | |
| 0-6 months |  |  |  |  |  |  | |  | |  | |  | |  | |  | |  | |  | |  | |  | |  | |
| Male | No partner bereavement | 200 | 20341.4 | 9.83 (8.56-11.29) | 1.00 | 1.00 | | 1.00 | | 0.38 | |  | |  | |  | |  | |  | |  | |  | |  | |
|  | Partner bereavement | 230 | 20243.8 | 11.36 (9.98-12.93) | 1.16 (0.96-1.40) | 1.18 (0.97-1.44) | | 1.15 (0.94-1.42) | |  |  |  | |  | |  | |  | |  | |  | |  | |  | |
| Female | No partner bereavement | 297 | 39121 | 7.59 (6.78-8.51) | 1.00 | 1.00 | | 1.00 | |  |  |  | |  | |  | |  | |  | |  | |  | |  | |
|  | Partner bereavement | 396 | 39109 | 10.13 (9.18-11.17) | 1.33 (1.15-1.55) | 1.32 (1.13-1.54) | | 1.30 (1.10-1.54) | |  |  |  | |  | |  | |  | |  | |  | |  | |  | |
| 0-12 months |  |  |  |  |  |  | |  | |  | |  | |  | |  | |  | |  | |  | |  | |  | |
| Male | No partner bereavement | 368 | 38999.4 | 9.44 (8.52-10.45) | 1.00 | 1.00 | | 1.00 | | 0.37 | |  | |  | |  | |  | |  | |  | |  | |  | |
|  | Partner bereavement | 383 | 38785.2 | 9.87 (8.93-10.92) | 1.05 (0.91-1.21) | 1.03 (0.89-1.20) | | 1.01 (0.87-1.18) | |  |  |  | |  | |  | |  | |  | |  | |  | |  | |
| Female | No partner bereavement | 580 | 75201.4 | 7.71 (7.11-8.37) | 1.00 | 1.00 | | 1.00 | |  |  |  | |  | |  | |  | |  | |  | |  | |  | |
|  | Partner bereavement | 672 | 75411.3 | 8.91 (8.26-9.61) | 1.16 (1.03-1.29) | 1.13 (1.00-1.27) | | 1.11 (0.98-1.25) | |  |  |  | |  | |  | |  | |  | |  | |  | |  | |
| 0-2 years |  |  |  |  |  |  | |  | |  | |  | |  | |  | |  | |  | |  | |  | |  | |
| Male | No partner bereavement | 643 | 71925.8 | 8.94 (8.27-9.66) | 1.00 | 1.00 | | 1.00 | | 1.00 | |  | |  | |  | |  | |  | |  | |  | |  | |
|  | Partner bereavement | 637 | 71551.6 | 8.90 (8.24-9.62) | 0.99 (0.89-1.11) | 0.99 (0.87-1.11) | | 0.95 (0.84-1.08) | |  |  |  | |  | |  | |  | |  | |  | |  | |  | |
| Female | No partner bereavement | 1075 | 138859 | 7.74 (7.29-8.22) | 1.00 | 1.00 | | 1.00 | |  |  |  | |  | |  | |  | |  | |  | |  | |  | |
|  | Partner bereavement | 1163 | 140681.6 | 8.27 (7.81-8.76) | 1.07 (0.98-1.16) | 1.04 (0.95-1.14) | | 1.02 (0.92-1.12) | |  |  |  | |  | |  | |  | |  | |  | |  | |  | |
| 0-5 years |  |  |  |  |  |  | |  | |  | |  | |  | |  | |  | |  | |  | |  | |  | |
| Male | No partner bereavement | 1350 | 141552.9 | 9.54 (9.04-10.06) | 1.00 | 1.00 | | 1.00 | | 0.20 | |  | |  | |  | |  | |  | |  | |  | |  | |
|  | Partner bereavement | 1287 | 141114.3 | 9.12 (8.64-9.63) | 0.95 (0.88-1.03) | 0.90 (0.82-0.99) | | 0.87 (0.79-0.96) | |  |  |  | |  | |  | |  | |  | |  | |  | |  | |
| Female | No partner bereavement | 2199 | 274461.7 | 8.01 (7.68-8.35) | 1.00 | 1.00 | | 1.00 | |  |  |  | |  | |  | |  | |  | |  | |  | |  | |
|  | Partner bereavement | 2389 | 287725.8 | 8.30 (7.98-8.64) | 1.03 (0.98-1.10) | 0.97 (0.91-1.04) | | 0.94 (0.88-1.01) | |  |  |  | |  | |  | |  | |  | |  | |  | |  | |
| Complete follow-up | |  |  |  |  |  | |  | |  | |  | |  | |  | |  | |  | |  | |  | |  | |
| Male | No partner bereavement | 2121 | 207950.8 | 10.20 (9.77-10.64) | 1.00 | 1.00 | | 1.00 | | 0.34 | |  | |  | |  | |  | |  | |  | |  | |  | |
|  | Partner bereavement | 2114 | 207379.8 | 10.19 (9.77-10.64) | 1.00 (0.94-1.06) | 0.93 (0.86-1.01) | | 0.91 (0.83-0.98) | |  |  |  | |  | |  | |  | |  | |  | |  | |  | |
| Female | No partner bereavement | 3576 | 405851.6 | 8.81 (8.53-9.10) | 1.00 | 1.00 | | 1.00 | |  |  |  | |  | |  | |  | |  | |  | |  | |  | |
|  | Partner bereavement | 4406 | 451520.5 | 9.76 (9.47-10.05) | 1.09 (1.05-1.14) | 0.97 (0.92-1.03) | | 0.95 (0.90-1.01) | |  |  |  | |  | |  | |  | |  | |  | |  | |  | |
|  |  |  |  |  |  |  | |  | |  | |  | |  | |  | |  | |  | |  | |  | |  | |
| **Age at bereavement (years)** | | |  |  |  |  | |  | |  | |  | |  | |  | |  | |  | |  | |  | |  | |
| 0-3 months |  |  |  |  |  |  | |  | |  | |  | |  | |  | |  | |  | |  | |  | |  | |
| 30-64 | No partner bereavement | 1 | 6208.8 | 0.16 (0.02-1.14) | 1.00 | 1.00 | | 1.00 | | 0.31 | |  | |  | |  | |  | |  | |  | |  | |  | |
|  | Partner bereavement | 1 | 6209 | 0.16 (0.02-1.14) | 1.00 (0.06-15.98) | 1.16 (0.07-18.74) | | 0.68 (0.04-11.87) | |  |  |  | |  | |  | |  | |  | |  | |  | |  | |
| 65-74 | No partner bereavement | 28 | 9950.3 | 2.81 (1.94-4.08) | 1.00 | 1.00 | | 1.00 | |  |  |  | |  | |  | |  | |  | |  | |  | |  | |
|  | Partner bereavement | 33 | 9942.9 | 3.32 (2.36-4.67) | 1.18 (0.71-1.95) | 1.19 (0.71-1.99) | | 1.14 (0.66-1.99) | |  |  |  | |  | |  | |  | |  | |  | |  | |  | |
| 75-84 | No partner bereavement | 119 | 11127.1 | 10.69 (8.94-12.80) | 1.00 | 1.00 | | 1.00 | |  |  |  | |  | |  | |  | |  | |  | |  | |  | |
|  | Partner bereavement | 205 | 11095.7 | 18.48 (16.11-21.19) | 1.73 (1.38-2.17) | 1.70 (1.35-2.14) | | 1.67 (1.31-2.13) | |  |  |  | |  | |  | |  | |  | |  | |  | |  | |
| ≥85 | No partner bereavement | 106 | 3050.8 | 34.75 (28.72-42.03) | 1.00 | 1.00 | | 1.00 | |  | |  | |  | |  | |  | |  | |  | |  | |  | |
|  | Partner bereavement | 128 | 3040.3 | 42.10 (35.40-50.06) | 1.21 (0.94-1.57) | 1.25 (0.95-1.64) | | 1.22 (0.92-1.63) | |  |  |  | |  | |  | |  | |  | |  | |  | |  | |
| 0-6 months |  |  |  |  |  |  | |  | |  | |  | |  | |  | |  | |  | |  | |  | |  | |
| 30-64 | No partner bereavement | 1 | 12285.7 | 0.08 (0.01-0.58) | 1.00 | 1.00 | | 1.00 | | 0.42 | |  | |  | |  | |  | |  | |  | |  | |  | |
|  | Partner bereavement | 4 | 12282.3 | 0.33 (0.12-0.87) | 4.00 (0.45-35.78) | 4.07 (0.45-36.75) | | 2.42 (0.25-23.07) | |  |  |  | |  | |  | |  | |  | |  | |  | |  | |
| 65-74 | No partner bereavement | 44 | 19596.9 | 2.25 (1.67-3.02) | 1.00 | 1.00 | | 1.00 | |  |  |  | |  | |  | |  | |  | |  | |  | |  | |
|  | Partner bereavement | 63 | 19570.8 | 3.22 (2.51-4.12) | 1.43 (0.98-2.11) | 1.42 (0.96-2.11) | | 1.41 (0.93-2.14) | |  |  |  | |  | |  | |  | |  | |  | |  | |  | |
| 75-84 | No partner bereavement | 243 | 21735.6 | 11.18 (9.86-12.68) | 1.00 | 1.00 | | 1.00 | |  |  |  | |  | |  | |  | |  | |  | |  | |  | |
|  | Partner bereavement | 333 | 21679.6 | 15.36 (13.80-17.10) | 1.37 (1.17-1.62) | 1.34 (1.13-1.59) | | 1.33 (1.11-1.59) | |  |  |  | |  | |  | |  | |  | |  | |  | |  | |
| ≥85 | No partner bereavement | 209 | 5844.2 | 35.76 (31.23-40.95) | 1.00 | 1.00 | | 1.00 | |  | |  | |  | |  | |  | |  | |  | |  | |  | |
|  | Partner bereavement | 226 | 5820.1 | 38.83 (34.08-44.24) | 1.09 (0.90-1.31) | 1.11 (0.91-1.36) | | 1.09 (0.89-1.34) | |  |  |  | |  | |  | |  | |  | |  | |  | |  | |
| 0-12 months |  |  |  |  |  |  | |  | |  | |  | |  | |  | |  | |  | |  | |  | |  | |
| 30-64 | No partner bereavement | 8 | 24051.5 | 0.33 (0.17-0.67) | 1.00 | 1.00 | | 1.00 | | 0.57 | |  | |  | |  | |  | |  | |  | |  | |  | |
|  | Partner bereavement | 9 | 24021.5 | 0.37 (0.19-0.72) | 1.13 (0.43-2.92) | 1.26 (0.47-3.40) | | 0.85 (0.31-2.31) | |  |  |  | |  | |  | |  | |  | |  | |  | |  | |
| 65-74 | No partner bereavement | 97 | 37972.4 | 2.55 (2.09-3.12) | 1.00 | 1.00 | | 1.00 | |  |  |  | |  | |  | |  | |  | |  | |  | |  | |
|  | Partner bereavement | 109 | 37951.2 | 2.87 (2.38-3.47) | 1.12 (0.85-1.48) | 1.07 (0.80-1.42) | | 1.09 (0.81-1.46) | |  |  |  | |  | |  | |  | |  | |  | |  | |  | |
| 75-84 | No partner bereavement | 479 | 41450.4 | 11.56 (10.57-12.64) | 1.00 | 1.00 | | 1.00 | |  |  |  | |  | |  | |  | |  | |  | |  | |  | |
|  | Partner bereavement | 558 | 41456.1 | 13.46 (12.39-14.62) | 1.17 (1.03-1.32) | 1.15 (1.01-1.31) | | 1.13 (0.99-1.30) | |  |  |  | |  | |  | |  | |  | |  | |  | |  | |
| ≥85 | No partner bereavement | 364 | 10726.5 | 33.93 (30.62-37.61) | 1.00 | 1.00 | | 1.00 | |  | |  | |  | |  | |  | |  | |  | |  | |  | |
|  | Partner bereavement | 379 | 10767.7 | 35.20 (31.83-38.93) | 1.04 (0.90-1.20) | 1.01 (0.87-1.19) | | 0.98 (0.84-1.16) | |  |  |  | |  | |  | |  | |  | |  | |  | |  | |
| 0-2 years |  |  |  |  |  |  | |  | |  | |  | |  | |  | |  | |  | |  | |  | |  | |
| 30-64 | No partner bereavement | 12 | 46111.5 | 0.26 (0.15-0.46) | 1.00 | 1.00 | | 1.00 | | 0.39 | |  | |  | |  | |  | |  | |  | |  | |  | |
|  | Partner bereavement | 15 | 45906.3 | 0.33 (0.20-0.54) | 1.26 (0.59-2.68) | 1.47 (0.66-3.27) | | 1.09 (0.48-2.45) | |  |  |  | |  | |  | |  | |  | |  | |  | |  | |
| 65-74 | No partner bereavement | 186 | 71268.4 | 2.61 (2.26-3.01) | 1.00 | 1.00 | | 1.00 | |  |  |  | |  | |  | |  | |  | |  | |  | |  | |
|  | Partner bereavement | 208 | 71390.4 | 2.91 (2.54-3.34) | 1.12 (0.92-1.36) | 1.12 (0.91-1.38) | | 1.08 (0.87-1.35) | |  |  |  | |  | |  | |  | |  | |  | |  | |  | |
| 75-84 | No partner bereavement | 917 | 75267.1 | 12.18 (11.42-13.00) | 1.00 | 1.00 | | 1.00 | |  |  |  | |  | |  | |  | |  | |  | |  | |  | |
|  | Partner bereavement | 971 | 76186.7 | 12.75 (11.97-13.57) | 1.05 (0.96-1.15) | 1.03 (0.93-1.13) | | 1.01 (0.91-1.12) | |  |  |  | |  | |  | |  | |  | |  | |  | |  | |
| ≥85 | No partner bereavement | 603 | 18137.8 | 33.25 (30.70-36.01) | 1.00 | 1.00 | | 1.00 | |  | |  | |  | |  | |  | |  | |  | |  | |  | |
|  | Partner bereavement | 606 | 18749.9 | 32.32 (29.85-35.00) | 0.97 (0.87-1.09) | 0.95 (0.83-1.08) | | 0.93 (0.81-1.06) | |  |  |  | |  | |  | |  | |  | |  | |  | |  | |
| 0-5 years |  |  |  |  |  |  | |  | |  | |  | |  | |  | |  | |  | |  | |  | |  | |
| 30-64 | No partner bereavement | 42 | 100785.4 | 0.42 (0.31-0.56) | 1.00 | 1.00 | | 1.00 | | 0.40 | |  | |  | |  | |  | |  | |  | |  | |  | |
|  | Partner bereavement | 45 | 99162.9 | 0.45 (0.34-0.61) | 1.09 (0.72-1.66) | 1.17 (0.75-1.84) | | 1.01 (0.64-1.60) | |  |  |  | |  | |  | |  | |  | |  | |  | |  | |
| 65-74 | No partner bereavement | 477 | 146584.2 | 3.25 (2.97-3.56) | 1.00 | 1.00 | | 1.00 | |  |  |  | |  | |  | |  | |  | |  | |  | |  | |
|  | Partner bereavement | 533 | 148745.9 | 3.58 (3.29-3.90) | 1.10 (0.97-1.25) | 1.03 (0.89-1.18) | | 0.99 (0.86-1.15) | |  |  |  | |  | |  | |  | |  | |  | |  | |  | |
| 75-84 | No partner bereavement | 2021 | 140315.3 | 14.40 (13.79-15.05) | 1.00 | 1.00 | | 1.00 | |  |  |  | |  | |  | |  | |  | |  | |  | |  | |
|  | Partner bereavement | 2102 | 148959.8 | 14.11 (13.52-14.73) | 0.98 (0.92-1.04) | 0.95 (0.88-1.02) | | 0.92 (0.86-1.00) | |  |  |  | |  | |  | |  | |  | |  | |  | |  | |
| ≥85 | No partner bereavement | 1009 | 28329.7 | 35.62 (33.49-37.88) | 1.00 | 1.00 | | 1.00 | |  | |  | |  | |  | |  | |  | |  | |  | |  | |
|  | Partner bereavement | 996 | 31971.4 | 31.15 (29.28-33.15) | 0.87 (0.80-0.95) | 0.88 (0.79-0.98) | | 0.85 (0.76-0.96) | |  |  |  | |  | |  | |  | |  | |  | |  | |  | |
| Complete follow-up | |  |  |  |  |  | |  | |  | |  | |  | |  | |  | |  | |  | |  | |  | |
| 30-64 | No partner bereavement | 142 | 177079.6 | 0.80 (0.68-0.95) | 1.00 | 1.00 | | 1.00 | | 0.05 | |  | |  | |  | |  | |  | |  | |  | |  | |
|  | Partner bereavement | 163 | 173884.9 | 0.94 (0.80-1.09) | 1.17 (0.93-1.46) | 1.32 (0.98-1.76) | | 1.22 (0.90-1.64) | |  |  |  | |  | |  | |  | |  | |  | |  | |  | |
| 65-74 | No partner bereavement | 1243 | 224441.3 | 5.54 (5.24-5.85) | 1.00 | 1.00 | | 1.00 | |  |  |  | |  | |  | |  | |  | |  | |  | |  | |
|  | Partner bereavement | 1493 | 238868.6 | 6.25 (5.94-6.58) | 1.12 (1.04-1.21) | 1.03 (0.93-1.14) | | 1.00 (0.90-1.11) | |  |  |  | |  | |  | |  | |  | |  | |  | |  | |
| 75-84 | No partner bereavement | 3153 | 181262.5 | 17.39 (16.80-18.01) | 1.00 | 1.00 | | 1.00 | |  |  |  | |  | |  | |  | |  | |  | |  | |  | |
|  | Partner bereavement | 3645 | 208668.8 | 17.47 (16.91-18.04) | 0.99 (0.94-1.04) | 0.95 (0.89-1.01) | | 0.93 (0.87-1.00) | |  |  |  | |  | |  | |  | |  | |  | |  | |  | |
| ≥85 | No partner bereavement | 1159 | 31019 | 37.36 (35.27-39.58) | 1.00 | 1.00 | | 1.00 | |  | |  | |  | |  | |  | |  | |  | |  | |  | |
|  | Partner bereavement | 1219 | 37478 | 32.53 (30.75-34.40) | 0.86 (0.79-0.93) | 0.87 (0.78-0.97) | | 0.84 (0.76-0.94) | |  |  |  | |  | |  | |  | |  | |  | |  | |  | |
|  |  |  |  |  |  |  | |  | |  | |  | |  | |  | |  | |  | |  | |  | |  | |
| **Calendar Year** | |  |  |  |  |  | |  | |  | |  | |  | |  | |  | |  | |  | |  | |  | |
| 0-3 months |  |  |  |  |  |  | |  | |  | |  | |  | |  | |  | |  | |  | |  | |  | |
| 1997-2004 | No partner bereavement | 58 | 8667.8 | 6.69 (5.17-8.66) | 1.00 | 1.00 | | 1.00 | | 0.08 | |  | |  | |  | |  | |  | |  | |  | |  | |
|  | Partner bereavement | 54 | 8654.1 | 6.24 (4.78-8.15) | 0.93 (0.64-1.35) | 1.00 (0.69-1.46) | | 0.96 (0.64-1.44) | |  |  |  | |  | |  | |  | |  | |  | |  | |  | |
| 2005-2009 | No partner bereavement | 65 | 10022.8 | 6.49 (5.09-8.27) | 1.00 | 1.00 | | 1.00 | |  |  |  | |  | |  | |  | |  | |  | |  | |  | |
|  | Partner bereavement | 117 | 10002.7 | 11.70 (9.76-14.02) | 1.80 (1.33-2.44) | 1.87 (1.37-2.56) | | 1.70 (1.23-2.37) | |  |  |  | |  | |  | |  | |  | |  | |  | |  | |
| 2010- | No partner bereavement | 131 | 11646.3 | 11.25 (9.48-13.35) | 1.00 | 1.00 | | 1.00 | |  |  |  | |  | |  | |  | |  | |  | |  | |  | |
|  | Partner bereavement | 196 | 11631.1 | 16.85 (14.65-19.38) | 1.50 (1.20-1.87) | 1.46 (1.16-1.83) | | 1.51 (1.17-1.93) | |  |  |  | |  | |  | |  | |  | |  | |  | |  | |
| 0-6 months |  |  |  |  |  |  | |  | |  | |  | |  | |  | |  | |  | |  | |  | |  | |
| 1997-2004 | No partner bereavement | 92 | 16715.1 | 5.50 (4.49-6.75) | 1.00 | 1.00 | | 1.00 | | 0.05 | |  | |  | |  | |  | |  | |  | |  | |  | |
|  | Partner bereavement | 83 | 16684.2 | 4.97 (4.01-6.17) | 0.90 (0.67-1.22) | 0.92 (0.68-1.24) | | 0.89 (0.64-1.22) | |  |  |  | |  | |  | |  | |  | |  | |  | |  | |
| 2005-2009 | No partner bereavement | 135 | 19763.9 | 6.83 (5.77-8.09) | 1.00 | 1.00 | | 1.00 | |  |  |  | |  | |  | |  | |  | |  | |  | |  | |
|  | Partner bereavement | 187 | 19733 | 9.48 (8.21-10.94) | 1.39 (1.11-1.73) | 1.46 (1.16-1.84) | | 1.43 (1.13-1.82) | |  |  |  | |  | |  | |  | |  | |  | |  | |  | |
| 2010- | No partner bereavement | 270 | 22983.3 | 11.75 (10.43-13.24) | 1.00 | 1.00 | | 1.00 | |  |  |  | |  | |  | |  | |  | |  | |  | |  | |
|  | Partner bereavement | 356 | 22935.5 | 15.52 (13.99-17.22) | 1.32 (1.13-1.55) | 1.29 (1.09-1.52) | | 1.28 (1.07-1.52) | |  |  |  | |  | |  | |  | |  | |  | |  | |  | |
| 0-12 months |  |  |  |  |  |  | |  | |  | |  | |  | |  | |  | |  | |  | |  | |  | |
| 1997-2004 | No partner bereavement | 158 | 31053 | 5.09 (4.35-5.95) | 1.00 | 1.00 | | 1.00 | | 0.03 | |  | |  | |  | |  | |  | |  | |  | |  | |
|  | Partner bereavement | 131 | 31030.7 | 4.22 (3.56-5.01) | 0.83 (0.66-1.05) | 0.79 (0.62-1.00) | | 0.79 (0.62-1.01) | |  |  |  | |  | |  | |  | |  | |  | |  | |  | |
| 2005-2009 | No partner bereavement | 268 | 38437.1 | 6.97 (6.19-7.86) | 1.00 | 1.00 | | 1.00 | |  |  |  | |  | |  | |  | |  | |  | |  | |  | |
|  | Partner bereavement | 315 | 38446.2 | 8.19 (7.34-9.15) | 1.17 (1.00-1.38) | 1.19 (1.00-1.41) | | 1.14 (0.96-1.36) | |  |  |  | |  | |  | |  | |  | |  | |  | |  | |
| 2010- | No partner bereavement | 522 | 44710.6 | 11.68 (10.72-12.72) | 1.00 | 1.00 | | 1.00 | |  |  |  | |  | |  | |  | |  | |  | |  | |  | |
|  | Partner bereavement | 609 | 44719.6 | 13.62 (12.58-14.74) | 1.17 (1.04-1.31) | 1.14 (1.01-1.30) | | 1.13 (0.99-1.29) | |  |  |  | |  | |  | |  | |  | |  | |  | |  | |
| 0-2 years |  |  |  |  |  |  | |  | |  | |  | |  | |  | |  | |  | |  | |  | |  | |
| 1997-2004 | No partner bereavement | 256 | 53408.4 | 4.79 (4.24-5.42) | 1.00 | 1.00 | | 1.00 | | 0.10 | |  | |  | |  | |  | |  | |  | |  | |  | |
|  | Partner bereavement | 215 | 53640.1 | 4.01 (3.51-4.58) | 0.84 (0.70-1.00) | 0.83 (0.68-1.00) | | 0.82 (0.67-0.99) | |  |  |  | |  | |  | |  | |  | |  | |  | |  | |
| 2005-2009 | No partner bereavement | 496 | 72287.8 | 6.86 (6.28-7.49) | 1.00 | 1.00 | | 1.00 | |  |  |  | |  | |  | |  | |  | |  | |  | |  | |
|  | Partner bereavement | 522 | 72755.1 | 7.17 (6.58-7.82) | 1.05 (0.92-1.18) | 1.05 (0.92-1.19) | | 1.01 (0.88-1.16) | |  |  |  | |  | |  | |  | |  | |  | |  | |  | |
| 2010- | No partner bereavement | 966 | 85088.5 | 11.35 (10.66-12.09) | 1.00 | 1.00 | | 1.00 | |  |  |  | |  | |  | |  | |  | |  | |  | |  | |
|  | Partner bereavement | 1063 | 85838 | 12.38 (11.66-13.15) | 1.09 (1.00-1.19) | 1.06 (0.96-1.17) | | 1.04 (0.93-1.15) | |  |  |  | |  | |  | |  | |  | |  | |  | |  | |
| 0-5 years |  |  |  |  |  |  | |  | |  | |  | |  | |  | |  | |  | |  | |  | |  | |
| 1997-2004 | No partner bereavement | 416 | 85705.3 | 4.85 (4.41-5.34) | 1.00 | 1.00 | | 1.00 | | 0.35 | |  | |  | |  | |  | |  | |  | |  | |  | |
|  | Partner bereavement | 365 | 87338.8 | 4.18 (3.77-4.63) | 0.86 (0.75-0.99) | 0.84 (0.72-0.98) | | 0.84 (0.72-0.98) | |  |  |  | |  | |  | |  | |  | |  | |  | |  | |
| 2005-2009 | No partner bereavement | 1027 | 145749.2 | 7.05 (6.63-7.49) | 1.00 | 1.00 | | 1.00 | |  |  |  | |  | |  | |  | |  | |  | |  | |  | |
|  | Partner bereavement | 1054 | 150276.9 | 7.01 (6.60-7.45) | 1.00 (0.91-1.08) | 0.94 (0.85-1.03) | | 0.90 (0.81-0.99) | |  |  |  | |  | |  | |  | |  | |  | |  | |  | |
| 2010- | No partner bereavement | 2106 | 184560 | 11.41 (10.93-11.91) | 1.00 | 1.00 | | 1.00 | |  |  |  | |  | |  | |  | |  | |  | |  | |  | |
|  | Partner bereavement | 2257 | 191224.4 | 11.80 (11.33-12.30) | 1.03 (0.97-1.10) | 0.98 (0.91-1.05) | | 0.95 (0.88-1.02) | |  |  |  | |  | |  | |  | |  | |  | |  | |  | |
| Complete follow-up |  |  |  |  |  |  | |  | |  | |  | |  | |  | |  | |  | |  | |  | |  | |
| 1997-2004 | No partner bereavement | 451 | 91083.4 | 4.95 (4.51-5.43) | 1.00 | 1.00 | | 1.00 | | 0.28 | |  | |  | |  | |  | |  | |  | |  | |  | |
|  | Partner bereavement | 408 | 93123.8 | 4.38 (3.98-4.83) | 0.88 (0.77-1.01) | 0.86 (0.74-0.99) | | 0.85 (0.73-0.99) | |  |  |  | |  | |  | |  | |  | |  | |  | |  | |
| 2005-2009 | No partner bereavement | 1431 | 196776.6 | 7.27 (6.91-7.66) | 1.00 | 1.00 | | 1.00 | |  |  |  | |  | |  | |  | |  | |  | |  | |  | |
|  | Partner bereavement | 1521 | 208026.9 | 7.31 (6.95-7.69) | 1.00 (0.93-1.08) | 0.94 (0.86-1.03) | | 0.91 (0.83-1.00) | |  |  |  | |  | |  | |  | |  | |  | |  | |  | |
| 2010- | No partner bereavement | 3815 | 325942.4 | 11.70 (11.34-12.08) | 1.00 | 1.00 | | 1.00 | |  |  |  | |  | |  | |  | |  | |  | |  | |  | |
|  | Partner bereavement | 4591 | 357749.6 | 12.83 (12.47-13.21) | 1.10 (1.05-1.14) | 0.98 (0.93-1.05) | | 0.96 (0.90-1.03) | |  |  |  | |  | |  | |  | |  | |  | |  | |  | |
|  |  |  |  |  |  |  | |  | |  | |  | |  | |  | |  | |  | |  | |  | |  | |
| **Manner of death (expected/unexpected, by assessing risk of death using Charlson score)** | | | | |  |  | |  | |  | |  | |  | |  | |  | |  | |  | |  | |  | |
| 0-3 months |  |  |  |  |  |  | |  | |  | |  | |  | |  | |  | |  | |  | |  | |  | |
| No partner bereavement | | 254 | 30337 | 8.37 (7.40-9.47) | 1.00 | 1.00 | | 1.00 | |  | |  | |  | |  | |  | |  | |  | |  | |  | |
| Low risk | | 94 | 19832.3 | 4.74 (3.87-5.80) | 0.58 (0.46-0.74) | 1.37 (1.00-1.88) | | 1.26 (0.90-1.77) | |  | |  | |  | |  | |  | |  | |  | |  | |  | |
| Medium risk | | 198 | 7764.9 | 25.50 (22.18-29.31) | 2.96 (2.46-3.56) | 1.47 (1.17-1.84) | | 1.42 (1.12-1.81) | |  | |  | |  | |  | |  | |  | |  | |  | |  | |
| High risk | | 75 | 2690.8 | 27.87 (22.23-34.95) | 2.90 (2.24-3.77) | 1.57 (1.08-2.28) | | 1.72 (1.16-2.56) | |  | |  | |  | |  | |  | |  | |  | |  | |  | |
| 0-6 months |  |  |  |  |  |  | |  | |  | |  | |  | |  | |  | |  | |  | |  | |  | |
| No partner bereavement | | 497 | 59462.4 | 8.36 (7.65-9.13) | 1.00 | 1.00 | | 1.00 | |  | |  | |  | |  | |  | |  | |  | |  | |  | |
| Low risk | | 162 | 39047.4 | 4.15 (3.56-4.84) | 0.52 (0.43-0.62) | 1.28 (1.00-1.63) | | 1.21 (0.94-1.56) | |  | |  | |  | |  | |  | |  | |  | |  | |  | |
| Medium risk | | 318 | 15107.1 | 21.05 (18.86-23.50) | 2.44 (2.12-2.81) | 1.20 (1.01-1.42) | | 1.17 (0.98-1.39) | |  | |  | |  | |  | |  | |  | |  | |  | |  | |
| High risk | | 146 | 5198.2 | 28.09 (23.88-33.03) | 2.85 (2.36-3.43) | 1.43 (1.10-1.85) | | 1.48 (1.13-1.95) | |  | |  | |  | |  | |  | |  | |  | |  | |  | |
| 0-12 months |  |  |  |  |  |  | |  | |  | |  | |  | |  | |  | |  | |  | |  | |  | |
| No partner bereavement | | 948 | 114200.8 | 8.30 (7.79-8.85) | 1.00 | 1.00 | | 1.00 | |  | |  | |  | |  | |  | |  | |  | |  | |  | |
| Low risk | | 285 | 75748.1 | 3.76 (3.35-4.23) | 0.47 (0.41-0.54) | 1.06 (0.89-1.26) | | 1.02 (0.85-1.23) | |  | |  | |  | |  | |  | |  | |  | |  | |  | |
| Medium risk | | 549 | 28709.9 | 19.12 (17.59-20.79) | 2.23 (2.01-2.48) | 1.09 (0.95-1.24) | | 1.07 (0.93-1.22) | |  | |  | |  | |  | |  | |  | |  | |  | |  | |
| High risk | | 221 | 9738.5 | 22.69 (19.89-25.89) | 2.31 (1.99-2.68) | 1.16 (0.94-1.44) | | 1.16 (0.93-1.44) | |  | |  | |  | |  | |  | |  | |  | |  | |  | |
| 0-2 years |  |  |  |  |  |  | |  | |  | |  | |  | |  | |  | |  | |  | |  | |  | |
| No partner bereavement | | 1718 | 210784.8 | 8.15 (7.77-8.55) | 1.00 | 1.00 | | 1.00 | |  | |  | |  | |  | |  | |  | |  | |  | |  | |
| Low risk | | 516 | 142793.9 | 3.61 (3.31-3.94) | 0.46 (0.42-0.51) | 1.01 (0.89-1.15) | | 0.98 (0.86-1.12) | |  | |  | |  | |  | |  | |  | |  | |  | |  | |
| Medium risk | | 933 | 52216.8 | 17.87 (16.76-19.05) | 2.12 (1.96-2.30) | 1.02 (0.92-1.13) | | 1.00 (0.90-1.11) | |  | |  | |  | |  | |  | |  | |  | |  | |  | |
| High risk | | 351 | 17222.5 | 20.38 (18.36-22.63) | 2.11 (1.88-2.37) | 1.01 (0.85-1.20) | | 0.99 (0.83-1.18) | |  | |  | |  | |  | |  | |  | |  | |  | |  | |
| 0-5 years |  |  |  |  |  |  | |  | |  | |  | |  | |  | |  | |  | |  | |  | |  | |
| No partner bereavement | | 3549 | 416014.5 | 8.53 (8.25-8.82) | 1.00 | 1.00 | | 1.00 | |  | |  | |  | |  | |  | |  | |  | |  | |  | |
| Low risk | | 1252 | 298577.7 | 4.19 (3.97-4.43) | 0.50 (0.47-0.54) | 0.97 (0.88-1.06) | | 0.94 (0.85-1.03) | |  | |  | |  | |  | |  | |  | |  | |  | |  | |
| Medium risk | | 1820 | 99614.3 | 18.27 (17.45-19.13) | 2.08 (1.96-2.20) | 0.93 (0.86-1.01) | | 0.91 (0.84-0.99) | |  | |  | |  | |  | |  | |  | |  | |  | |  | |
| High risk | | 604 | 30648.1 | 19.71 (18.20-21.34) | 1.98 (1.82-2.16) | 0.93 (0.81-1.07) | | 0.89 (0.77-1.02) | |  | |  | |  | |  | |  | |  | |  | |  | |  | |
| Complete follow-up | |  |  |  |  |  | |  | |  | |  | |  | |  | |  | |  | |  | |  | |  | |
| No partner bereavement | | 5697 | 613802.4 | 9.28 (9.04-9.53) | 1.00 | 1.00 | | 1.00 | |  | |  | |  | |  | |  | |  | |  | |  | |  | |
| Low risk | | 2954 | 485758.9 | 6.08 (5.87-6.30) | 0.66 (0.63-0.69) | 1.01 (0.94-1.09) | | 0.99 (0.92-1.07) | |  | |  | |  | |  | |  | |  | |  | |  | |  | |
| Medium risk | | 2760 | 135387.7 | 20.39 (19.64-21.16) | 2.16 (2.07-2.26) | 0.92 (0.85-0.99) | | 0.90 (0.83-0.97) | |  | |  | |  | |  | |  | |  | |  | |  | |  | |
| High risk | | 806 | 37753.8 | 21.35 (19.92-22.87) | 2.03 (1.89-2.19) | 0.92 (0.80-1.04) | | 0.88 (0.77-1.00) | |  | |  | |  | |  | |  | |  | |  | |  | |  | |
|  |  |  |  |  |  |  | |  | |  | |  | |  | |  | |  | |  | |  | |  | |  | |
| **Living alone at time of bereavement** | |  |  |  |  |  | |  | |  | |  | |  | |  | |  | |  | |  | |  | |  | |
| 0-3 months |  |  |  |  |  |  | |  | |  | |  | |  | |  | |  | |  | |  | |  | |  | |
| No partner bereavement | | 254 | 30337 | 8.37 (7.40-9.47) | 1.00 | 1.00 | | 1.00 | |  | |  | |  | |  | |  | |  | |  | |  | |  | |
| Bereaved - not living alone | | 96 | 13643.1 | 7.04 (5.76-8.59) | 0.83 (0.65-1.05) | 1.43 (1.03-1.98) | | 1.47 (1.04-2.07) | |  | |  | |  | |  | |  | |  | |  | |  | |  | |
| Bereaved - living alone | | 271 | 16644.9 | 16.28 (14.45-18.34) | 1.97 (1.66-2.34) | 1.47 (1.21-1.78) | | 1.42 (1.16-1.74) | |  | |  | |  | |  | |  | |  | |  | |  | |  | |
| 0-6 months |  |  |  |  |  |  | |  | |  | |  | |  | |  | |  | |  | |  | |  | |  | |
| No partner bereavement | | 497 | 59462.4 | 8.36 (7.65-9.13) | 1.00 | 1.00 | | 1.00 | |  | |  | |  | |  | |  | |  | |  | |  | |  | |
| Bereaved - not living alone | | 183 | 26793.4 | 6.83 (5.91-7.89) | 0.80 (0.68-0.95) | 1.26 (1.00-1.59) | | 1.24 (0.98-1.58) | |  | |  | |  | |  | |  | |  | |  | |  | |  | |
| Bereaved - living alone | | 443 | 32559.4 | 13.61 (12.40-14.93) | 1.66 (1.46-1.88) | 1.27 (1.09-1.46) | | 1.24 (1.07-1.45) | |  | |  | |  | |  | |  | |  | |  | |  | |  | |
| 0-12 months |  |  |  |  |  |  | |  | |  | |  | |  | |  | |  | |  | |  | |  | |  | |
| No partner bereavement | | 948 | 114200.8 | 8.30 (7.79-8.85) | 1.00 | 1.00 | | 1.00 | |  | |  | |  | |  | |  | |  | |  | |  | |  | |
| Bereaved - not living alone | | 309 | 51744.9 | 5.97 (5.34-6.68) | 0.70 (0.62-0.80) | 1.06 (0.89-1.26) | | 1.02 (0.85-1.22) | |  | |  | |  | |  | |  | |  | |  | |  | |  | |
| Bereaved - living alone | | 746 | 62451.6 | 11.95 (11.12-12.83) | 1.46 (1.33-1.61) | 1.11 (0.99-1.24) | | 1.09 (0.97-1.22) | |  | |  | |  | |  | |  | |  | |  | |  | |  | |
| 0-2 years |  |  |  |  |  |  | |  | |  | |  | |  | |  | |  | |  | |  | |  | |  | |
| No partner bereavement | | 1718 | 210784.8 | 8.15 (7.77-8.55) | 1.00 | 1.00 | | 1.00 | |  | |  | |  | |  | |  | |  | |  | |  | |  | |
| Bereaved - not living alone | | 549 | 96636.5 | 5.68 (5.23-6.18) | 0.68 (0.62-0.75) | 0.95 (0.83-1.08) | | 0.92 (0.80-1.05) | |  | |  | |  | |  | |  | |  | |  | |  | |  | |
| Bereaved - living alone | | 1251 | 115596.7 | 10.82 (10.24-11.44) | 1.35 (1.25-1.45) | 1.05 (0.96-1.14) | | 1.03 (0.94-1.12) | |  | |  | |  | |  | |  | |  | |  | |  | |  | |
| 0-5 years |  |  |  |  |  |  | |  | |  | |  | |  | |  | |  | |  | |  | |  | |  | |
| No partner bereavement | | 3549 | 416014.5 | 8.53 (8.25-8.82) | 1.00 | 1.00 | | 1.00 | |  | |  | |  | |  | |  | |  | |  | |  | |  | |
| Bereaved - not living alone | | 1169 | 196667.5 | 5.94 (5.61-6.29) | 0.68 (0.64-0.73) | 0.92 (0.84-1.02) | | 0.89 (0.80-0.98) | |  | |  | |  | |  | |  | |  | |  | |  | |  | |
| Bereaved - living alone | | 2507 | 232172.6 | 10.80 (10.38-11.23) | 1.28 (1.22-1.35) | 0.96 (0.89-1.02) | | 0.93 (0.87-1.00) | |  | |  | |  | |  | |  | |  | |  | |  | |  | |
| Complete follow-up | |  |  |  |  |  | |  | |  | |  | |  | |  | |  | |  | |  | |  | |  | |
| No partner bereavement | | 5697 | 613802.4 | 9.28 (9.04-9.53) | 1.00 | 1.00 | | 1.00 | |  | |  | |  | |  | |  | |  | |  | |  | |  | |
| Bereaved - not living alone | | 2140 | 308184 | 6.94 (6.66-7.24) | 0.73 (0.69-0.77) | 0.92 (0.85-1.00) | | 0.89 (0.82-0.97) | |  | |  | |  | |  | |  | |  | |  | |  | |  | |
| Bereaved - living alone | | 4380 | 350716.3 | 12.49 (12.12-12.86) | 1.35 (1.30-1.41) | 0.98 (0.92-1.03) | | 0.96 (0.90-1.02) | |  | |  | |  | |  | |  | |  | |  | |  | |  | |
| OR, odds ratio. CI, confidence interval.  *Cox model with time in study timescale, and no stratification by matched set  **Cox model with age timescale, stratified by matched set, additionally adjusted for gender, calendar time and IMD  ^†^Additional adjustment for: BMI, smoking status, alcohol use, depression, diabetes, hearing loss, hypertension and carer status. | | | | | | | | |  | |  | |  | |  | |  | |  | |  | |  | |  | |  |
|  |  |  |  |  |  |  |  |  |  | |  | |  | |  | |  | |  | |  | |  | |  | |  |
|  |  |  |  |  |  |  |  |  |  | |  | |  | |  | |  | |  | |  | |  | |  | |  |
|  |  |  |  |  |  |  |  |  |  | |  | |  | |  | |  | |  | |  | |  | |  | |  |
|  |  |  |  |  |  |  |  | |  | |  | |  | |  | |  | |  | |  | |  | |  | |  |

| **Supplementary Table 3. Crude consultation rate by exposure group and time-period, and main analysis additionally updated for time-updated depression and time-updated diabetes.** | | | | | |  |
| --- | --- | --- | --- | --- | --- | --- |
|  |  |  |  |  |  | |
|  | **Median consultations* per year (IQR)** | **Number (%) of individuals with depression during follow-up** | **Additionally adjusted for time-updated depression** | **Crude incidence diabetes during follow-up** | **Additionally adjusted for time-updated diabetes** | |
| **Time since bereavement** |  |  |  |  |  | |
| *0-3 months* |  |  |  |  |  | |
| No partner bereavement | 12.0 ( 4.0-20.0) | 690 (0.6) | 1.00 | 11223 (9.1) | 1.00 | |
| Partner bereavement | 16.0 ( 8.0-24.0) | 1949 (1.6) | 1.43 (1.20-1.71) | 13151 (10.6) | 1.43 (1.20-1.71) | |
| *0-6 months* |  |  |  |  |  | |
| No partner bereavement | 12.0 ( 4.0-21.9) | 1277 (1.0) |  | 11365 (9.2) | 1.00 | |
| Partner bereavement | 14.0 ( 6.0-24.0) | 3641 (2.9) | 1.24 (1.09-1.41) | 13334 (10.8) | 1.25 (1.10-1.42) | |
| *0-12 months* |  |  |  |  |  | |
| No partner bereavement | 12.0 ( 6.0-21.0) | 1896 (1.5) | 1.00 | 11682 (9.4) | 1.00 | |
| Partner bereavement | 14.0 ( 7.0-24.0) | 5483 (4.4) | 1.06 (0.96-1.17) | 13587 (11.0) | 1.08 (0.98-1.19) | |
| *0-2 years* |  |  |  |  |  | |
| No partner bereavement | 12.5 ( 6.5-21.5) | 2731 (2.2) | 1.00 | 12013 (9.7) | 1.00 | |
| Partner bereavement | 14.0 ( 7.7-23.5) | 7350 (5.9) | 0.97 (0.90-1.05) | 14007 (11.3) | 1.00 (0.93-1.08) | |
| *0-5 years* |  |  |  |  |  | |
| No partner bereavement | 13.4 ( 7.4-22.6) | 3936 (3.2) | 1.00 | 12709 (10.3) | 1.00 | |
| Partner bereavement | 14.8 ( 8.4-24.4) | 9474 (7.7) | 0.88 (0.83-0.94) | 14815 (12.0) | 0.92 (0.87-0.97) | |
| Complete follow-up |  |  |  |  |  | |
| No partner bereavement | 14.4 ( 8.2-23.5) | 5074 (4.1) | 1.00 | 13347 (10.8) | 1.00 | |
| Partner bereavement | 15.9 ( 9.4-25.5) | 10895 (8.8) | 0.90 (0.85-0.94) | 15645 (12.6) | 0.93 (0.89-0.98) | |
| *Consultations included all face-to face contacts: total number of consultations was divided by total follow-up time and expressed as a per year consultation rate. | | | | | |  |

| **Supplementary Table 4. Main analyses repeated, varying the study criteria** | | | | | | |
| --- | --- | --- | --- | --- | --- | --- |
|  |  |  |  |  |  |  |
|  | **Number of events** | **Total person time (person-years)** | **Crude rate (per 1000 person-years)** | **Crude HR* (95% CI)** | **Minimally-adjusted HR** (95% CI)** | **Fully-adjusted HR† (95% CI)** |
| Excluding individuals with a history of dementia symptoms (N=232,516 ) | | |  |  |  |  |
| *0-3 months* |  |  |  |  |  |  |
| No partner bereavement | 122 | 29404.1 | 4.15 (3.47-4.95) | 1.00 | 1.00 | 1.00 |
| Partner bereavement | 183 | 29359.5 | 6.23 (5.39-7.20) | 1.50 (1.19-1.89) | 1.50 (1.18-1.90) | 1.42 (1.09-1.84) |
| *0-6 months* |  |  |  |  |  |  |
| No partner bereavement | 277 | 57714.6 | 4.80 (4.27-5.40) | 1.00 | 1.00 | 1.00 |
| Partner bereavement | 344 | 57636.1 | 5.97 (5.37-6.63) | 1.24 (1.06-1.46) | 1.26 (1.06-1.49) | 1.23 (1.03-1.47) |
| *0-12 months* |  |  |  |  |  |  |
| No partner bereavement | 593 | 111036.6 | 5.34 (4.93-5.79) | 1.00 | 1.00 | 1.00 |
| Partner bereavement | 647 | 111135.4 | 5.82 (5.39-6.29) | 1.09 (0.97-1.22) | 1.04 (0.92-1.17) | 0.99 (0.87-1.12) |
| *0-2 years* |  |  |  |  |  |  |
| No partner bereavement | 1178 | 205463.4 | 5.73 (5.42-6.07) | 1.00 | 1.00 | 1.00 |
| Partner bereavement | 1242 | 207094.6 | 6.00 (5.67-6.34) | 1.05 (0.97-1.13) | 1.01 (0.92-1.10) | 0.97 (0.88-1.06) |
| *0-5 years* |  |  |  |  |  |  |
| No partner bereavement | 2798 | 407364.2 | 6.87 (6.62-7.13) | 1.00 | 1.00 | 1.00 |
| Partner bereavement | 2973 | 420244.6 | 7.07 (6.82-7.33) | 1.03 (0.98-1.08) | 0.95 (0.89-1.02) | 0.91 (0.86-0.98) |
| Complete follow-up |  |  |  |  |  |  |
| No partner bereavement | 4880 | 603390.1 | 8.09 (7.86-8.32) | 1.00 | 1.00 | 1.00 |
| Partner bereavement | 5759 | 648177.6 | 8.88 (8.66-9.12) | 1.09 (1.05-1.13) | 0.96 (0.91-1.02) | 0.94 (0.89-0.99) |
|  |  |  |  |  |  |  |
| Restricting study start date to 1st April 2005 (N=171,332) | | |  |  |  |  |
| *0-3 months* |  |  |  |  |  |  |
| No partner bereavement | 191 | 20908.3 | 9.14 (7.93-10.53) | 1.00 | 1.00 | 1.00 |
| Partner bereavement | 304 | 20873.9 | 14.56 (13.02-16.30) | 1.59 (1.33-1.91) | 1.60 (1.32-1.93) | 1.60 (1.30-1.96) |
| *0-6 months* |  |  |  |  |  |  |
| No partner bereavement | 393 | 40802.4 | 9.63 (8.73-10.63) | 1.00 | 1.00 | 1.00 |
| Partner bereavement | 528 | 40724.2 | 12.97 (11.91-14.12) | 1.35 (1.18-1.53) | 1.35 (1.18-1.55) | 1.32 (1.14-1.53) |
| *0-12 months* |  |  |  |  |  |  |
| No partner bereavement | 762 | 77651.9 | 9.81 (9.14-10.54) | 1.00 | 1.00 | 1.00 |
| Partner bereavement | 878 | 77652.1 | 11.31 (10.58-12.08) | 1.15 (1.05-1.27) | 1.13 (1.02-1.26) | 1.11 (0.99-1.24) |
| *0-2 years* |  |  |  |  |  |  |
| No partner bereavement | 1368 | 140700 | 9.72 (9.22-10.25) | 1.00 | 1.00 | 1.00 |
| Partner bereavement | 1479 | 141697.4 | 10.44 (9.92-10.98) | 1.07 (1.00-1.15) | 1.05 (0.97-1.14) | 1.02 (0.94-1.12) |
| *0-5 years* |  |  |  |  |  |  |
| No partner bereavement | 2671 | 262315.8 | 10.18 (9.80-10.58) | 1.00 | 1.00 | 1.00 |
| Partner bereavement | 2846 | 270142.9 | 10.54 (10.16-10.93) | 1.03 (0.98-1.09) | 0.98 (0.92-1.05) | 0.95 (0.89-1.02) |
| Complete follow-up |  |  |  |  |  |  |
| No partner bereavement | 3529 | 330110.3 | 10.69 (10.34-11.05) | 1.00 | 1.00 | 1.00 |
| Partner bereavement | 3946 | 347780.6 | 11.35 (11.00-11.71) | 1.06 (1.01-1.11) | 0.98 (0.92-1.04) | 0.95 (0.89-1.01) |
|  |  |  |  |  |  |  |
| Requiring two dementia diagnoses on separate dates within one year (N=247,586) | | | |  |  |  |
| *0-3 months* |  |  |  |  |  |  |
| No partner bereavement | 47 | 30337 | 1.55 (1.16-2.06) | 1.00 | 1.00 | 1.00 |
| Partner bereavement | 60 | 30287.9 | 1.98 (1.54-2.55) | 1.28 (0.87-1.87) | 1.18 (0.77-1.79) | 0.97 (0.59-1.60) |
| *0-6 months* |  |  |  |  |  |  |
| No partner bereavement | 68 | 59462.4 | 1.14 (0.90-1.45) | 1.00 | 1.00 | 1.00 |
| Partner bereavement | 109 | 59352.8 | 1.84 (1.52-2.22) | 1.61 (1.19-2.17) | 1.56 (1.13-2.17) | 1.61 (1.12-2.32) |
| *0-12 months* |  |  |  |  |  |  |
| No partner bereavement | 122 | 114200.8 | 1.07 (0.89-1.28) | 1.00 | 1.00 | 1.00 |
| Partner bereavement | 158 | 114196.5 | 1.38 (1.18-1.62) | 1.29 (1.02-1.64) | 1.22 (0.94-1.57) | 1.21 (0.92-1.59) |
| *0-2 years* |  |  |  |  |  |  |
| No partner bereavement | 218 | 210784.8 | 1.03 (0.91-1.18) | 1.00 | 1.00 | 1.00 |
| Partner bereavement | 252 | 212233.2 | 1.19 (1.05-1.34) | 1.15 (0.96-1.38) | 1.09 (0.89-1.34) | 1.10 (0.88-1.37) |
| *0-5 years* |  |  |  |  |  |  |
| No partner bereavement | 440 | 416014.5 | 1.06 (0.96-1.16) | 1.00 | 1.00 | 1.00 |
| Partner bereavement | 487 | 428840.1 | 1.14 (1.04-1.24) | 1.07 (0.94-1.22) | 1.03 (0.88-1.21) | 1.01 (0.85-1.19) |
| Complete follow-up |  |  |  |  |  |  |
| No partner bereavement | 750 | 613802.4 | 1.22 (1.14-1.31) | 1.00 | 1.00 | 1.00 |
| Partner bereavement | 892 | 658900.3 | 1.35 (1.27-1.45) | 1.09 (0.99-1.21) | 1.01 (0.88-1.15) | 0.97 (0.85-1.12) |
|  |  |  |  |  |  |  |
| Restricting to patients with linked data only (N=89,926) | | |  |  |  |  |
| *0-3 months* |  |  |  |  |  |  |
| No partner bereavement | 24 | 11670.3 | 2.06 (1.38-3.07) | 1.00 | 1.00 | 1.00 |
| Partner bereavement | 48 | 12289.9 | 3.91 (2.94-5.18) | 1.91 (1.17-3.13) | 1.84 (1.09-3.11) | 2.22 (1.14-4.29) |
| *0-6 months* |  |  |  |  |  |  |
| No partner bereavement | 107 | 22989.4 | 4.65 (3.85-5.63) | 1.00 | 1.00 | 1.00 |
| Partner bereavement | 135 | 24224 | 5.57 (4.71-6.60) | 1.20 (0.93-1.55) | 1.58 (1.11-2.25) | 1.50 (1.01-2.24) |
| *0-12 months* |  |  |  |  |  |  |
| No partner bereavement | 256 | 44250.2 | 5.79 (5.12-6.54) | 1.00 | 1.00 | 1.00 |
| Partner bereavement | 275 | 46758.2 | 5.88 (5.23-6.62) | 1.02 (0.86-1.21) | 1.22 (0.94-1.59) | 1.16 (0.86-1.54) |
| *0-2 years* |  |  |  |  |  |  |
| No partner bereavement | 486 | 81757.5 | 5.94 (5.44-6.50) | 1.00 | 1.00 | 1.00 |
| Partner bereavement | 539 | 87084 | 6.19 (5.69-6.73) | 1.05 (0.93-1.18) | 1.06 (0.86-1.30) | 0.98 (0.79-1.22) |
| *0-5 years* |  |  |  |  |  |  |
| No partner bereavement | 1053 | 161227.6 | 6.53 (6.15-6.94) | 1.00 | 1.00 | 1.00 |
| Partner bereavement | 1209 | 176290 | 6.86 (6.48-7.26) | 1.05 (0.97-1.15) | 1.02 (0.87-1.18) | 0.93 (0.79-1.10) |
| Complete follow-up |  |  |  |  |  |  |
| No partner bereavement | 1712 | 238095.5 | 7.19 (6.86-7.54) | 1.00 | 1.00 | 1.00 |
| Partner bereavement | 2154 | 273885.9 | 7.86 (7.54-8.20) | 1.09 (1.02-1.16) | 1.06 (0.92-1.21) | 1.00 (0.87-1.15) |
| OR, odds ratio; CI, confidence interval. | | |  |  |  |  |
| *Cox model with time in study timescale, and no stratification by matched set | | | |  |  |  |
| **Cox model with age timescale, stratified by matched set, additionally adjusted for calendar time, IMD and time since study entry | | | | | |  |
| ^†^Additional adjustment for: BMI, smoking status, alcohol use, depression, diabetes, hearing loss, hypertension and carer status. | | | | | |  |

| **Supplementary Table 5. Alternative outcome analyses, where the outcome is acute cardiovascular events (stroke and myocardial infarction) in those without a history of an acute cardiovascular event (N=205,574)** | | | | | | | | | | | | |  |  |  |  |  |  |  |  |  |  |  |  |  |  |  |  |  |  |  |  |
| --- | --- | --- | --- | --- | --- | --- | --- | --- | --- | --- | --- | --- | --- | --- | --- | --- | --- | --- | --- | --- | --- | --- | --- | --- | --- | --- | --- | --- | --- | --- | --- | --- |
|  |  | | |  |  |  | |  | |  |  | | |  | | | | |  | | | |  | | | |  | | |  | | |
|  | | **Crude HR* (95% CI)** | **Minimally-adjusted HR** (95% CI)** | | | | | **Adjusted HR† (95% CI)** | | | |  | | |  | |  | | | |  | | | |  | | | |  |  |  |  |
| **Time since bereavement** | |  |  | | | | |  | | | |  | | |  | |  | | | |  | | | |  | | | |  |  |  |  |
| *0-3 months* | |  |  | | | | |  | | | |  | | |  | |  | | | |  | | | |  | | | |  |  |  |  |
| No partner bereavement | | 1.00 | 1.00 | | | | | 1.00 | | | |  | | |  | |  | | | |  | | | |  | | | |  |  |  |  |
| Partner bereavement | | 1.30 (1.10-1.54) | 1.31 (1.10-1.56) | | | | | 1.27 (1.06-1.53) | | | |  | | |  | |  | | | |  | | | |  | | | |  |  |  |  |
| *0-6 months* | |  |  | | | | |  | | | |  | | |  | |  | | | |  | | | |  | | | |  |  |  |  |
| No partner bereavement | | 1.00 | 1.00 | | | | | 1.00 | | | |  | | |  | |  | | | |  | | | |  | | | |  |  |  |  |
| Partner bereavement | | 1.14 (1.00-1.28) | 1.20 (1.05-1.36) | | | | | 1.15 (1.00-1.31) | | | |  | | |  | |  | | | |  | | | |  | | | |  |  |  |  |
| *0-12 months* | |  |  | | | | |  | | | |  | | |  | |  | | | |  | | | |  | | | |  |  |  |  |
| No partner bereavement | | 1.00 | 1.00 | | | | | 1.00 | | | |  | | |  | |  | | | |  | | | |  | | | |  |  |  |  |
| Partner bereavement | | 1.13 (1.04-1.24) | 1.20 (1.09-1.32) | | | | | 1.16 (1.05-1.28) | | | |  | | |  | |  | | | |  | | | |  | | | |  |  |  |  |
| *0-2 years* | |  |  | | | | |  | | | |  | | |  | |  | | | |  | | | |  | | | |  |  |  |  |
| No partner bereavement | | 1.00 | 1.00 | | | | | 1.00 | | | |  | | |  | |  | | | |  | | | |  | | | |  |  |  |  |
| Partner bereavement | | 1.10 (1.03-1.17) | 1.16 (1.08-1.25) | | | | | 1.12 (1.04-1.20) | | | |  | | |  | |  | | | |  | | | |  | | | |  |  |  |  |
| *0-5 years* | |  |  | | | | |  | | | |  | | |  | |  | | | |  | | | |  | | | |  |  |  |  |
| No partner bereavement | | 1.00 | 1.00 | | | | | 1.00 | | | |  | | |  | |  | | | |  | | | |  | | | |  |  |  |  |
| Partner bereavement | | 1.10 (1.05-1.14) | 1.13 (1.07-1.19) | | | | | 1.08 (1.02-1.14) | | | |  | | |  | |  | | | |  | | | |  | | | |  |  |  |  |
| Complete follow-up | |  |  | | | | |  | | | |  | | |  | |  | | | |  | | | |  | | | |  |  |  |  |
| No partner bereavement | | 1.00 | 1.00 | | | | | 1.00 | | | |  | | |  | |  | | | |  | | | |  | | | |  |  |  |  |
| Partner bereavement | | 1.13 (1.10-1.17) | 1.11 (1.06-1.16) | | | | | 1.06 (1.02-1.11) | | | |  | | |  | |  | | | |  | | | |  | | | |  |  |  |  |
| OR, odds ratio; CI, confidence interval. | | |  | | | | |  | | | |  | | |  | |  | | | |  | | | |  | | | |  |  |  |  |
| *Cox model with time in study timescale, and no stratification by matched set | | | | | | |  | | | | | |  | | |  | | | |  | | | |  | | | |  | | |  |  |
| **Cox model with age timescale, stratified by matched set, additionally adjusted for calendar time, IMD and time sine bereavement | | | | | | | | |  | | | | | | | | |  | | | |  | | | |  | | |  | | |  |
| ^†^Additional adjustment for: BMI, smoking status, alcohol use, depression, diabetes, hearing loss, hypertension and carer status. | | | | | | | | |  | | | | | | | | |  | | | |  | | | |  | | |  | | |  |

| **Supplementary Table 6. Crude rate for dementia diagnosis by exposure to partner bereavement, and unadjusted and adjusted hazard ratios, by partner illness.** | | | | | | | | | | | | | | | | | | | |  | | | |  | |  | |  | |  | |  |
| --- | --- | --- | --- | --- | --- | --- | --- | --- | --- | --- | --- | --- | --- | --- | --- | --- | --- | --- | --- | --- | --- | --- | --- | --- | --- | --- | --- | --- | --- | --- | --- | --- |
|  |  | | | |  | | |  | |  | | |  | | |  | | |  | |  | | | |  | |  | |  | |  | |
|  |  | | | | **Number of events** | | | **Total person time (person-years)** | | **Crude rate (per 1000 person-years)** | | | **Crude HR* (95% CI)** | | | **Minimally-adjusted HR** (95% CI)** | | | **Adjusted HR† (95% CI)** | |  | | | |  | |  | |  | |  | |
| **Partner had a terminal illness** | | | |  | |  | | |  | | |  | | |  | | |  | |  | | | |  | |  | |  | |  | |  |
| 0-3 months | | | |  | |  | | |  | | |  | | |  | | |  | |  | | | |  | |  | |  | |  | |  |
|  | No partner bereavement | | | | 254 | | | 30337 | | 8.37 (7.40-9.47) | | | 1.00 | | | 1.00 | | | 1.00 | |  | | | |  | |  | |  | |  | |
|  | Partner bereavement: not terminally ill | | | | 337 | | | 29090.5 | | 11.58 (10.41-12.89) | | | 1.39 (1.18-1.64) | | | 1.43 (1.20-1.70) | | | 1.38 (1.15-1.67) | |  | | | |  | |  | |  | |  | |
|  | Partner bereavement: terminally ill | | | | 30 | | | 1197.4 | | 25.05 (17.52-35.83) | | | 2.49 (1.70-3.64) | | | 1.93 (1.03-3.61) | | | 2.10 (1.08-4.06) | |  | | | |  | |  | |  | |  | |
|  |  | | | |  | | |  | |  | | |  | | |  | | |  | |  | | | |  | |  | |  | |  | |
| 0-6 months | | | |  | |  | | |  | | |  | | |  | | |  | |  | | | |  | |  | |  | |  | |  |
|  | No partner bereavement | | | | 497 | | | 59462.4 | | 8.36 (7.65-9.13) | | | 1.00 | | | 1.00 | | | 1.00 | |  | | | |  | |  | |  | |  | |
|  | Partner bereavement: not terminally ill | | | | 572 | | | 57040.8 | | 10.03 (9.24-10.88) | | | 1.21 (1.08-1.37) | | | 1.24 (1.09-1.41) | | | 1.21 (1.05-1.38) | |  | | | |  | |  | |  | |  | |
|  | Partner bereavement: terminally ill | | | | 54 | | | 2312 | | 23.36 (17.89-30.50) | | | 2.22 (1.67-2.94) | | | 1.63 (1.04-2.55) | | | 1.74 (1.08-2.78) | |  | | | |  | |  | |  | |  | |
|  |  | | | |  | | |  | |  | | |  | | |  | | |  | |  | | | |  | |  | |  | |  | |
| 0-12 months | | | |  | |  | | |  | | |  | | |  | | |  | |  | | | |  | |  | |  | |  | |  |
|  | No partner bereavement | | | | 948 | | | 114200.8 | | 8.30 (7.79-8.85) | | | 1.00 | | | 1.00 | | | 1.00 | |  | | | |  | |  | |  | |  | |
|  | Partner bereavement: not terminally ill | | | | 971 | | | 109865.6 | | 8.84 (8.30-9.41) | | | 1.08 (0.98-1.18) | | | 1.07 (0.97-1.18) | | | 1.05 (0.95-1.16) | |  | | | |  | |  | |  | |  | |
|  | Partner bereavement: terminally ill | | | | 84 | | | 4330.9 | | 19.40 (15.66-24.02) | | | 1.85 (1.48-2.32) | | | 1.36 (0.96-1.92) | | | 1.42 (0.99-2.03) | |  | | | |  | |  | |  | |  | |
|  |  | | | |  | | |  | |  | | |  | | |  | | |  | |  | | | |  | |  | |  | |  | |
| 0-2 years | | | |  | |  | | |  | | |  | | |  | | |  | |  | | | |  | |  | |  | |  | |  |
|  | No partner bereavement | | | | 1718 | | | 210784.8 | | 8.15 (7.77-8.55) | | | 1.00 | | | 1.00 | | | 1.00 | |  | | | |  | |  | |  | |  | |
|  | Partner bereavement: not terminally ill | | | | 1671 | | | 204600 | | 8.17 (7.78-8.57) | | | 1.01 (0.95-1.08) | | | 1.00 (0.93-1.08) | | | 0.98 (0.90-1.05) | |  | | | |  | |  | |  | |  | |
|  | Partner bereavement: terminally ill | | | | 129 | | | 7633.2 | | 16.90 (14.22-20.08) | | | 1.64 (1.37-1.97) | | | 1.24 (0.93-1.64) | | | 1.27 (0.95-1.70) | |  | | | |  | |  | |  | |  | |
|  |  | | | |  | | |  | |  | | |  | | |  | | |  | |  | | | |  | |  | |  | |  | |
| 0-5 years | | | |  | |  | | |  | | |  | | |  | | |  | |  | | | |  | |  | |  | |  | |  |
|  | No partner bereavement | | | | 3549 | | | 416014.5 | | 8.53 (8.25-8.82) | | | 1.00 | | | 1.00 | | | 1.00 | |  | | | |  | |  | |  | |  | |
|  | Partner bereavement: not terminally ill | | | | 3461 | | | 415463.8 | | 8.33 (8.06-8.61) | | | 0.98 (0.94-1.03) | | | 0.94 (0.89-0.99) | | | 0.91 (0.86-0.96) | |  | | | |  | |  | |  | |  | |
|  | Partner bereavement: terminally ill | | | | 215 | | | 13376.3 | | 16.07 (14.06-18.37) | | | 1.53 (1.33-1.75) | | | 1.09 (0.87-1.37) | | | 1.09 (0.86-1.38) | |  | | | |  | |  | |  | |  | |
|  |  | | | |  | | |  | |  | | |  | | |  | | |  | |  | | | |  | |  | |  | |  | |
| Complete follow-up | | | |  | |  | | |  | | |  | | |  | | |  | |  | | | |  | |  | |  | |  | |  |
|  | No partner bereavement | | | | 5697 | | | 613802.4 | | 9.28 (9.04-9.53) | | | 1.00 | | | 1.00 | | | 1.00 | |  | | | |  | |  | |  | |  | |
|  | Partner bereavement: not terminally ill | | | | 6235 | | | 642596.1 | | 9.70 (9.46-9.95) | | | 1.04 (1.00-1.08) | | | 0.96 (0.91-1.00) | | | 0.93 (0.89-0.98) | |  | | | |  | |  | |  | |  | |
|  | Partner bereavement: terminally ill | | | | 285 | | | 16304.2 | | 17.48 (15.56-19.63) | | | 1.59 (1.41-1.79) | | | 1.01 (0.82-1.26) | | | 1.01 (0.81-1.26) | |  | | | |  | |  | |  | |  | |
| OR, odds ratio; CI, confidence interval. | | | |  | |  | | |  | | |  | | |  | | |  | |  | | | |  | |  | |  | |  | |  |
| *Cox model with time in study timescale, and no stratification by matched set | | | | | | | | |  | | |  | | |  | | |  | |  | | | |  | |  | |  | |  | |  |
| **Cox model with age timescale, stratified by matched set, additionally adjusted for gender, calendar time and IMD | | | | | | | | | | | |  | | |  | | |  | |  | | | |  | |  | |  | |  | |  |
| ^†^Additional adjustment for: BMI, smoking status, alcohol use, depression, diabetes, hearing loss, hypertension and carer status. | | | | | | | | | | | |  | | |  | | |  | |  | | | |  | |  | |  | |  | |  |
|  |  | | | |  | | |  | |  | | |  | | |  | | |  | |  | | | |  | |  | |  | |  | |
|  |  | | | |  | | |  | |  | | |  | | |  | | |  | |  | | | |  | |  | |  | |  | |
|  |  | | | |  | | |  | |  | | |  | | |  | | |  | |  | | | |  | |  | |  | |  | |
|  |  | | | |  | | |  | |  | | |  | | |  | | |  | |  | | | |  | |  | |  | |  | |
|  |  | | | |  | | |  | |  | | |  | | |  | | |  | |  | | | |  | |  | |  | |  | |
|  |  | | | |  | | |  | |  | | |  | | |  | | |  | |  | | | |  | |  | |  | |  | |
|  |  | | | |  | | |  | |  | | |  | | |  | | |  | |  | | | |  | |  | |  | |  | |
|  |  | | | |  | | |  | |  | | |  | | |  | | |  | |  | | | |  | |  | |  | |  | |
|  |  | | | |  | | |  | |  | | |  | | |  | | |  | |  | | | |  | |  | |  | |  | |
|  |  | | | |  | | |  | |  | | |  | | |  | | |  | |  | | | |  | |  | |  | |  | |
|  |  | | | |  | | |  | |  | | |  | | |  | | |  | |  | | | |  | |  | |  | |  | |
|  |  | | | |  | | |  | |  | | |  | | |  | | |  | |  | | | |  | |  | |  | |  | |
|  |  | | | |  | | |  | |  | | |  | | |  | | |  | |  | | | |  | |  | |  | |  | |
|  |  | | | |  | | |  | |  | | |  | | |  | | |  | |  | | | |  | |  | |  | |  | |
|  |  | | | |  | | |  | |  | | |  | | |  | | |  | |  | | | |  | |  | |  | |  | |
|  |  | | | |  | | |  | |  | | |  | | |  | | |  | |  | | | |  | |  | |  | |  | |
|  |  | | | |  | | |  | |  | | |  | | |  | | |  | |  | | | |  | |  | |  | |  | |
|  |  | | | |  | | |  | |  | | |  | | |  | | |  | |  | | | |  | |  | |  | |  | |
|  |  | | | |  | | |  | |  | | |  | | |  | | |  | |  | | | |  | |  | |  | |  | |
|  |  | | | |  | | |  | |  | | |  | | |  | | |  | |  | | | |  | |  | |  | |  | |
| **Supplementary Table 7. Crude rate for dementia diagnosis by exposure to partner bereavement, and unadjusted and adjusted hazard ratios, by discrete time periods since bereavement.** | | | | | | | | | | | | | | | | | | | | | | | |  |  |  |  |  |  |  |  |  |
|  | | |  |  | | | |  | | | |  | | |  | | |  | |  | | |  |  |  |  |  |  |  |  |  |  |
|  | | |  | **Number of events** | | | | **Total person time (person-years)** | | | | **Crude rate (per 1000 person-years)** | | | **Crude HR* (95% CI)** | | | **Minimally-adjusted HR** (95% CI)** | | **Adjusted HR^†^ (95% CI)** | | |  |  |  |  |  |  |  |  |  |  |
| Time period | | |  |  | | | |  | | | |  | | |  | | |  | |  | | |  |  |  |  |  |  |  |  |  |  |
| 0-3 months | | | No partner bereavement | 254 | | | | 30337 | | | | 8.37 (7.40-9.47) | | | 1.00 | | | 1.00 | | 1.00 | | |  |  |  |  |  |  |  |  |  |  |
|  | | | Partner bereavement | 367 | | | | 30287.9 | | | | 12.12 (10.94-13.42) | | | 1.45 (1.23-1.70) | | | 1.46 (1.24-1.72) | | 1.43 (1.20-1.71) | | |  |  |  |  |  |  |  |  |  |  |
| 3-6 months | | | No partner bereavement | 243 | | | | 29125.4 | | | | 8.34 (7.36-9.46) | | | 1.00 | | | 1.00 | | 1.00 | | |  |  |  |  |  |  |  |  |  |  |
|  | | | Partner bereavement | 259 | | | | 29064.8 | | | | 8.91 (7.89-10.07) | | | 1.07 (0.90-1.27) | | | 1.04 (0.87-1.26) | | 1.05 (0.86-1.28) | | |  |  |  |  |  |  |  |  |  |  |
| 6-12 months | | | No partner bereavement | 451 | | | | 54738.5 | | | | 8.24 (7.51-9.04) | | | 1.00 | | | 1.00 | | 1.00 | | |  |  |  |  |  |  |  |  |  |  |
|  | | | Partner bereavement | 429 | | | | 54843.7 | | | | 7.82 (7.12-8.60) | | | 0.95 (0.83-1.08) | | | 0.89 (0.77-1.03) | | 0.87 (0.75-1.02) | | |  |  |  |  |  |  |  |  |  |  |
| 1-2 years | | | No partner bereavement | 770 | | | | 96584 | | | | 7.97 (7.43-8.56) | | | 1.00 | | | 1.00 | | 1.00 | | |  |  |  |  |  |  |  |  |  |  |
|  | | | Partner bereavement | 745 | | | | 98036.7 | | | | 7.60 (7.07-8.16) | | | 0.95 (0.86-1.05) | | | 0.91 (0.81-1.02) | | 0.87 (0.77-0.99) | | |  |  |  |  |  |  |  |  |  |  |
| 2-5 years | | | No partner bereavement | 1831 | | | | 205229.8 | | | | 8.92 (8.52-9.34) | | | 1.00 | | | 1.00 | | 1.00 | | |  |  |  |  |  |  |  |  |  |  |
|  | | | Partner bereavement | 1876 | | | | 216606.9 | | | | 8.66 (8.28-9.06) | | | 0.97 (0.91-1.03) | | | 0.86 (0.79-0.93) | | 0.82 (0.75-0.89) | | |  |  |  |  |  |  |  |  |  |  |
| >5 years | | | No partner bereavement | 2148 | | | | 197787.8 | | | | 10.86 (10.41-11.33) | | | 1.00 | | | 1.00 | | 1.00 | | |  |  |  |  |  |  |  |  |  |  |
|  | | | Partner bereavement | 2844 | | | | 230060.2 | | | | 12.36 (11.92-12.82) | | | 1.13 (1.07-1.20) | | | 1.00 (0.90-1.10) | | 0.99 (0.90-1.10) | | |  |  |  |  |  |  |  |  |  |  |
| HR, hazards ratio; CI, confidence interval. | | | |  | | | |  | | | |  | | |  | | |  | |  | | |  |  |  |  |  |  |  |  |  |  |
| *Cox model with time in study timescale, and no stratification by matched set | | | | | | | | | | | |  | | |  | | |  | |  | | |  |  |  |  |  |  |  |  |  |  |
| **Cox model with age timescale, stratified by matched set, additionally adjusted for calendar time and IMD | | | | | | | | | | | | | | |  | | |  | |  | | |  |  |  |  |  |  |  |  |  |  |
| ^†^Additional adjustment for: BMI, smoking status, alcohol use, depression, diabetes, hearing loss, hypertension and carer status. | | | | | | | | | | | | | | | | | |  | |  | | |  |  |  |  |  |  |  |  |  |  |
